# Early non-invasive ventilation and high-flow nasal oxygen therapy for preventing endotracheal intubation in hypoxemic blunt chest trauma patients: the OptiTHO randomized trial

**DOI:** 10.1186/s13054-023-04429-2

**Published:** 2023-04-26

**Authors:** Cédric Carrié, Benjamin Rieu, Antoine Benard, Kilian Trin, Laurent Petit, Alexandre Massri, Igor Jurcison, Guillaume Rousseau, David Tran Van, Marie Reynaud Salard, Jeremy Bourenne, Albrice Levrat, Laurent Muller, Damien Marie, Claire Dahyot-Fizelier, Julien Pottecher, Jean-Stéphane David, Thomas Godet, Matthieu Biais

**Affiliations:** 1 Surgical and Trauma Intensive Care Unit, Anesthesiology and Critical Care Department, Hôpital Pellegrin, CHU Bordeaux, Bordeaux University Hospital, Place Amélie Raba Léon, 33076 Bordeaux Cedex, France; 2 Anesthesiology and Critical Care Department, Clermont – Ferrand University Hospital, Clermont – Ferrand, France; 3 Pôle de Santé Publique, Service d’information Médicale, Clinical Epidemiology Unit (USMR), CHU Bordeaux, Bordeaux, France; 4 Anesthesiology and Critical Care Department, Pau Hospital, Pau, France; 5 Anesthesiology and Critical Care Department, Beaujon University Hospital, Paris, France; 6 Anesthesiology and Critical Care Department, Robert Picqué Hospital, Bordeaux, France; 7 Anesthesiology and Critical Care Department, Saint Etienne University Hospital, Saint Etienne, France; 8 Emergency and Critical Care Department, Hôpital de La Timone, Marseille University Hospital, Marseille, France; 9 Anesthesiology and Critical Care Department, Annecy Hospital, Annecy, France; 10 Anesthesiology and Critical Care Department, Nimes University Hospital, Nimes, France; 11 Anesthesiology and Critical Care Department, Poitiers University Hospital, Poitiers, France; 12 Anesthesiology and Critical Care Department, Strasbourg University Hospital, Strasbourg, France; 13 Department of Anesthesia and Intensive Care, Groupe Hospitalier Sud, Hospices Civils de Lyon (HCL), Lyon, France; 14 Research On Healthcare Performance (RESHAPE), INSERM U1290, University Claude Bernard Lyon 1, Lyon, France; 15 INSERM U1034, Biology of Cardiovascular Diseases, Bordeaux University, Pessac, France

**Keywords:** Chest trauma, Respiratory failure, Non-invasive ventilation, High-flow nasal oxygen therapy, Intensive care

## Abstract

**Background:**

The benefit–risk ratio of prophylactic non-invasive ventilation (NIV) and high-flow nasal oxygen therapy (HFNC-O_2_) during the early stage of blunt chest trauma remains controversial because of limited data. The main objective of this study was to compare the rate of endotracheal intubation between two NIV strategies in high-risk blunt chest trauma patients.

**Methods:**

The OptiTHO trial was a randomized, open-label, multicenter trial over a two-year period. Every adult patients admitted in intensive care unit within 48 h after a high-risk blunt chest trauma (Thoracic Trauma Severity Score ≥ 8), an estimated PaO_2_/FiO_2_ ratio < 300 and no evidence of acute respiratory failure were eligible for study enrollment (Clinical Trial Registration: NCT03943914). The primary objective was to compare the rate of endotracheal intubation for delayed respiratory failure between two NIV strategies: i) a prompt association of HFNC-O_2_ and “early” NIV in every patient for at least 48 h with vs. ii) the standard of care associating COT and “late” NIV, indicated in patients with respiratory deterioration and/or PaO_2_/FiO_2_ ratio ≤ 200 mmHg. Secondary outcomes were the occurrence of chest trauma-related complications (pulmonary infection, delayed hemothorax or moderate-to-severe ARDS).

**Results:**

Study enrollment was stopped for futility after a 2-year study period and randomization of 141 patients. Overall, 11 patients (7.8%) required endotracheal intubation for delayed respiratory failure. The rate of endotracheal intubation was not significantly lower in patients treated with the experimental strategy (7% [5/71]) when compared to the control group (8.6% [6/70]), with an adjusted OR = 0.72 (95%IC: 0.20–2.43), *p* = *0.60*. The occurrence of pulmonary infection, delayed hemothorax or delayed ARDS was not significantly lower in patients treated by the experimental strategy (adjusted OR = 1.99 [95%IC: 0.73–5.89], *p* = *0.18,* 0.85 [95%IC: 0.33–2.20], *p* = *0.74* and 2.14 [95%IC: 0.36–20.77], *p* = *0.41*, respectively).

**Conclusion:**

A prompt association of HFNC-O_2_ with preventive NIV did not reduce the rate of endotracheal intubation or secondary respiratory complications when compared to COT and late NIV in high-risk blunt chest trauma patients with non-severe hypoxemia and no sign of acute respiratory failure.

*Clinical Trial Registration*: NCT03943914, Registered 7 May 2019.

**Supplementary Information:**

The online version contains supplementary material available at 10.1186/s13054-023-04429-2.

## Background

In blunt chest trauma patients with no immediate life-threatening injuries, non-invasive ventilation (NIV) has been widely used to prevent endotracheal intubation although the actual benefits have not been fully documented. To date, the literature in the field has been impaired by large heterogeneity in study designs, patients’ severity (i.e., acute respiratory failure or severe hypoxemia) or difference in comparators (i.e., oxygen or invasive ventilation), precluding strong recommendations regarding the most appropriate time for NIV initiation [1–5]. Given the positive overall results, current guidelines support the cautious use of NIV to prevent intubation in appropriately selected patients with hypoxemic respiratory failure (defined as having PaO_2_/FiO_2_ < 200 mmHg) [6, 7].

The benefit–risk ratio of early NIV, before the occurrence of respiratory distress or severe hypoxemia in blunt chest trauma patients, remains controversial because of limited data [8]. Moreover, high-flow nasal oxygen therapy (HFNC-O_2_) appears to be a reliable and better tolerated alternative to conventional oxygen therapy (COT), associated with a significant reduction in intubation rate in patients with acute hypoxemic respiratory failure [9]. In this regard, we hypothesized that a prompt non-invasive respiratory support (association of HFNC-O_2_ with preventive NIV) may prevent the risk of chest trauma-related respiratory complications through the maintenance of alveolar recruitment, delivery of fully conditioned gas and reduction in ventilatory drive [10].

The main objective of this multicenter, randomized, controlled trial was to compare the rate of endotracheal intubation within 14 days after randomization between two NIV strategies in high-risk blunt chest trauma patients with an estimated PaO_2_/FiO_2_ ratio ≤ 300 mmHg and no evidence of acute respiratory failure: a preventive strategy, associating HFNC-O_2_ and early NIV vs. the recommended standard-of-care, associating COT and late NIV in patients with a PaO_2_/FiO_2_ ratio ≤ 200 mmHg or delayed respiratory failure.

## Methods

### Study design, population and settings

The OptiTHO trial was a randomized, open-label, multicenter trial involving 12 centers over a two-year period, from September 2019 to September 2021 (ClinicalTrials.gov Identifier: NCT03943914). The trial was approved for all centers by the *Comité de Protection des Personnes Ile-de-France* (IRB number: 2019 – A00532 – 55). Written informed consent was obtained from the patients or their relatives in all cases. Trial execution was monitored by an independent data and safety committee.

Patients were eligible for study enrollment if they met all of the following criteria: adult patients admitted in intensive care unit (ICU) within 48 h after a high-risk blunt chest trauma, with a thoracic trauma severity score (TTSS) ≥ 8 and an estimated PaO_2_/FiO_2_ ratio < 300 under oxygen therapy (oxygen flow [L/min] × 0.3 + 0.21) [11]. Non-inclusion criteria were as follows: need for emergency intubation (*see criteria below*), hypercapnia (PaCO_2_ > 45 mmHg) and/or exacerbation of underlying cardiorespiratory disease, previous surgical intervention by thoracotomy or laparotomy, contra-indications for NIV (such as Glasgow Coma Scale score ≤ 12 or excessive agitation, hemodynamic instability, complex facial trauma, tracheobronchial or esophageal injuries…), a *do-not-intubate* order or a decision not to participate. Details of the study inclusion and exclusion criteria are provided in the Additional file 1: Appendix.

Randomization was performed using a secured centralized web-based management system with stratification by center and PaO_2_/FiO_2_ value, in a 1:1 ratio to one of the following strategies:

- A preventive strategy, associating HFNC-O_2_ and “early” NIV in patients with an estimated PaO_2_/FiO_2_ ratio ≤ 300 mmHg (HFNC-O_2_ + early NIV)
- The standard of care associating COT and “late” NIV, indicated in patients with signs of acute respiratory failure and/or with an estimated PaO_2_/FiO_2_ ratio ≤ 200 mmHg (COT ± late NIV)

### Study interventions

In the preventive strategy (HFNC-O_2_ + early NIV), NIV was started immediately after inclusion regardless of the PaO_2_/FiO_2_ ratio. The minimum required duration of non-invasive ventilation was 4 h per day for at least 2 calendar days. Between NIV sessions, HFNC-O_2_ was administered continuously through a nasal cannula, with FiO_2_ and gas flow rate adjusted simultaneously to maintain a SpO2 > 92% or PaO_2_ > 65 mmHg. Beyond the first 48 h, HFNC-O_2_ and NIV could be stopped and the patient switched to COT if respiratory rate < 25/min and SpO_2_ > 92% under FiO_2_ < 30% for at least 6 h.

In the control group (COT ± late NIV), COT was initially administered from nasal cannula or high concentration oxygen mask, according to the oxygen supply needed to achieve a SpO_2_ > 92%. The secondary introduction of NIV was initiated in patients with PaO_2_/FiO_2_ ratio < 200 mmHg under COT and/or who developed signs of acute respiratory failure with no other organ dysfunction.

In both strategies, NIV was delivered with an ICU ventilator through the best tolerated interface (nasal mask, face mask or helmet if available). Pressure support was titrated to achieve an expired tidal volume of 7 to 10 ml/kg of predicted body weight with a respiratory rate < 25/min. Positive end expiratory pressure (PEEP) was initially set at 5 cmH_2_O and then gradually increased to obtain a minimal FiO_2_ while minimizing leaks and patients’ discomfort. The daily duration of NIV could be extended at the discretion of the physician in patients with evidence of acute respiratory failure under COT or HFNC-O_2_ and improving under NIV.

During the first 48 h, patient’s tolerance and blood gas analysis (PaO_2_/FiO_2_ ratio, PaCO_2_) were recorded every 6 h under each respiratory device (COT, HFNC-O_2_ or NIV). All other aspects of patients’ clinical management were in accordance with the up-to-date recommendations [6]. Unless contraindicated, the standard treatment included locoregional procedure (epidural analgesia or alternative techniques unless contraindication), prompt mobilization and physiotherapy, surgical advice for flail chest management or retained hemothorax.

The summary figure of the protocol is resumed in Additional file 1.

### Study outcomes

The main study outcome was the need for endotracheal intubation for respiratory failure within 14 days after randomization and/or end-of-hospitalization. To ensure consistency of indications between sites and to reduce the risk of delayed intubation, the following criteria for endotracheal intubation were used: cardiac arrest or significant hemodynamic instability, worsening of neurologic status, acute respiratory failure defined by at least two of the following criteria: respiratory rate ≥ 35 / min, high respiratory-muscle workload, abundant tracheal secretions, signs of respiratory exhaustion (pH < 7.32 or PaCO_2_ > 50 mmHg) and/or severe hypoxemia (PaO_2_/FiO_2_ ratio < 100 or SpO_2_ < 92% for more than 5 min) [5, 9].

A rescue NIV trial was allowed at the discretion of the physician in patients with acute respiratory failure and no other organ dysfunction. The persistence of worsening of acute respiratory failure or severe hypoxemia after 1 h of NIV or in patients with NIV-intolerance were considered as criteria for endotracheal intubation [12]. The NIV-dependence (defined as the resumption of acute respiratory failure or severe hypoxemia under COT or HFNC-O_2_ with need for continuous NIV ≥ 12 consecutive hours) was also considered as criteria for endotracheal intubation. For patients requiring emergency or scheduled surgery after randomization, endotracheal intubation for general anesthesia was not considered a failure of the NIV strategy, provided that the patient could be weaned from the mechanical ventilation within 8 h postoperatively. An independent adjudication committee was responsible for validating the consistency of endotracheal intubations based on clinical, biological and imaging data, blinded from the randomization group.

Secondary outcomes were the time-course PaO_2_/FiO_2_ ratio and PaCO_2_ over the first 48 h in each patient, the occurrence of chest trauma—related complications (occurrence of pulmonary infection, delayed hemothorax with need for chest tube insertion or moderate-to-severe ARDS in accordance with the Berlin definition [13]), the occurrence of potential NIV side effects (pneumothorax, vomiting/aspiration, excessive agitation with need for sedatives or NIV removal), the ICU and hospital length of stay, the in-hospital mortality within 14 days after randomization and/or end-of-hospitalization. A complete definition of secondary outcome is given in Additional file 1.

### Statistical analysis

An intention-to-treat analysis was performed as the principal analysis. The primary outcome was compared between groups by using a logistic regression model adjusted on randomization stratification factors (center and PaO_2_/FiO_2_ ratio at inclusion). For the secondary outcome, a mixed effect model was constructed to determine the association of the NIV strategies with PaO_2_/FiO_2_ and PaCO_2_ variations over time. The conditions of validity of mixed effect and linear regression models (normal distribution and homoscedasticity of residuals) were systematically checked.

Sample size calculation was based on an estimated rate of the primary endpoint of 12% in the experimental group and 25% in the control group, in accordance with previous studies in high-risk trauma patients with TTS score ≥ 8 [14, 15]. A sample size of 278 patients (139 patients per group) was required to provide more than 80% power to show the superiority of the preventive strategy vs. the standard-of-care using a χ^2^ test with a two-sided type I error rate of 5%.

However, the intubation rate was much lower than expected. Over-estimation of incidence of the primary outcome made the study likely underpowered to detect any inter-group difference, even if the computed sample size was reached. Consequently, study enrollment was stopped for futility after a 2-year study period and randomization of 141 patients.

Statistical analyses were performed by the Clinical Epidemiology Unit (USMR, Bordeaux University Hospital) with the R software (version 4.2.1).

## Results

**Fig. 1:**
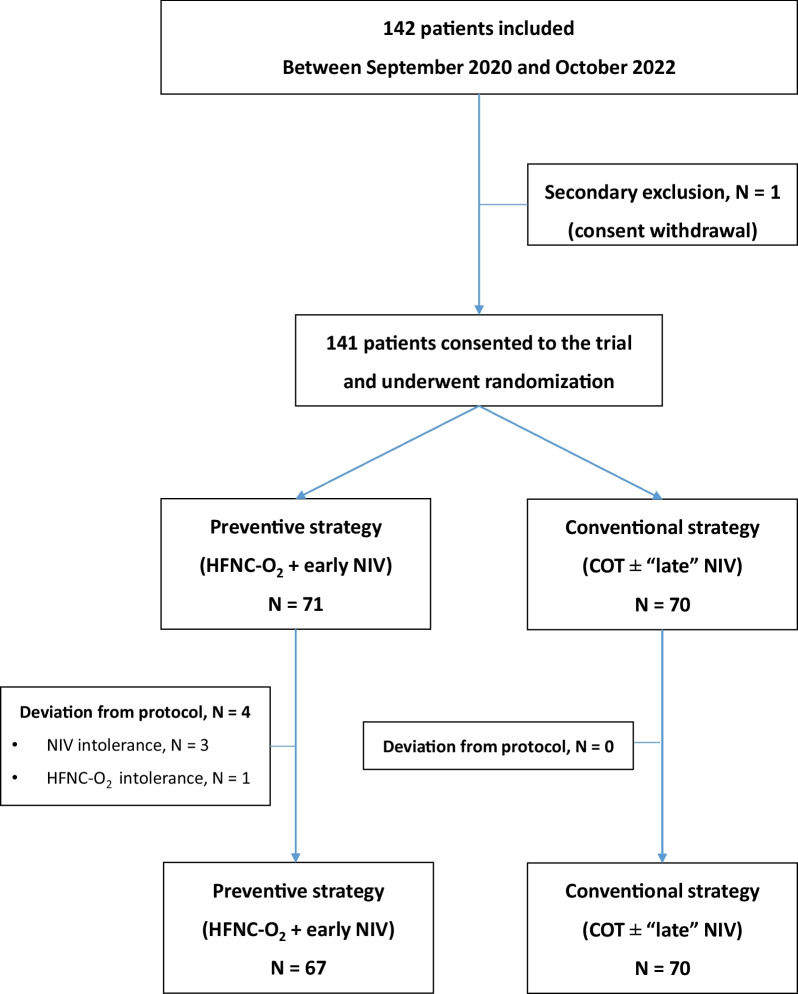
Study flowchart

During the 2-year study period of inclusion, 141 patients were randomized in the 12 participating centers: 71 were treated with the preventive strategy (HFNC-O_2_ + early NIV) and 70 patients were assigned to the control group (COT ± late NIV). In this subgroup of patients, 44 (63%) received COT only and 26 (37%) received an associated NIV (17 [24%] for deterioration of the PaO_2_/FiO_2_ ratio < 200 and 9 [13%] for other signs of isolated respiratory failure). The median duration of NIV was 8 [6–16] hours, with mean expired tidal volume of 8.3 ± 0.6 ml/kg and FiO_2_ of 34 ± 9%. The median duration of HFNC-O_2_ was 40 [38–53] hours, with mean flow rate of 37 ± 9 L/min and FiO2 of 35 ± 10%. The study flowchart is depicted Fig. 1. The characteristics of the population are resumed Table 1.

**Table 1:** Main characteristics of the population

	Overall population	HFNC-O_2_ + early NIV	COT ± late NIV
	*N* = 141	*N* = 71	*N* = 70
Demographics and medical history			
Age (years)	60 [50–70]	60 [48–72]	60 [52–69]
Male sex	120 (85)	58 (82)	62 (89)
BMI (kg/m^2^)	26 [24–29]	26 [24–29]	27 [25–30]
Preexisting respiratory conditions*	24 (17)	15 (21)	9 (13)
Antiplatelet or anticoagulant treatment	26 (18)	11 (15)	15 (21)
Thoracic injuries			
Number of rib fractures	7 [5–9]	7 [5–9]	8 [6–9]
Flail chest	66 (47)	28 (39)	38 (54)
Pulmonary contusion	98 (70)	47 (66)	51 (73)
Pneumothorax	93 (66)	42 (59)	51 (73)
Hemothorax	82 (58)	38 (54)	44 (63)
Associated injuries (AIS ≥ 3)			
Limb fracture	48 (34)	27 (38)	21 (30)
Craniofacial trauma	24 (17)	14 (20)	10 (14)
Pelvic fracture	24 (17)	13 (18)	11 (16)
Spine fracture	16 (11)	6 (9)	10 (14)
Abdominal trauma	12 (9)	7 (10)	5 (7)
Severity scores			
ISS	25 [17–34]	26 [17–34]	24 [17–33]
TTSS	11 [9–13]	11 [9–12]	12 [10–13]
Time between trauma and randomization	17 [10–29]	17 [12–29]	17 [9–28]
Clinical and biological data at randomization			
Respiratory rate	18 [15–21]	18 [15–22]	17 [15–20]
PaO_2_ (mmHg)	74 [65–81]	72 [66–79]	74 [66–84]
Oxygen flow rate (L/min)	3 [2–4]	3 [2–4]	3 [2–4]
Estimated PaO_2_/FiO_2_	258 [222–278]	254 [220–279]	260 [226–276]
PaCO_2_ (mmHg)	39 [37–42]	39 [36–42]	40 [37–42]
Chest pain scale at rest	3 [2–5]	3 [2–5]	3 [2–5]
ICU management within 14 days after randomization			
Need for non-invasive ventilation	97 (69)	71 (100)	26 (37)
Need for emergency or scheduled surgery	32 (23)	17 (24)	15 (21)
Rib fixation or other thoracic surgery	12 (9)	3 (4)	9 (13)
Non-thoracic surgery	24 (17)	15 (21)	9 (13)
Need for locoregional procedure	81 (57)	37 (52)	44 (63)
Need for epidural analgesia	51 (36)	21 (30)	30 (43)
Need for alternative techniques only	30 (21)	16 (23)	14 (20)
Need for chest tube insertion	48 (34)	22 (31)	26 (37)
Patient’s outcome			
Need for intubation for delayed respiratory failure	11 (8)	5 (7)	6 (9)
Occurrence of pulmonary infection	22 (16)	15 (21)	7 (10)
Delayed hemothorax with need for chest tube insertion	21 (15)	11 (16)	10 (14)
Moderate-to-severe ARDS	8 (6)	5 (7)	3 (4)
In-hospital mortality	3 (2)	2 (3)	1 (1)
ICU length of stay (days)	6 [4–9]	6 [4–9]	6 [4–9]
Hospital length of stay (days)	11 [8–18]	12 [7–21]	11 [9–15]

Results expressed as number (percentage) or median [interquartile 25–75%]. AIS: Abbreviated Injury Score; ARDS: Acute Respiratory Distress Syndrome [15]; BMI: Body Mass Index; ICU: Intensive Care Unit; COT: Conventional Oxygen Therapy; HFNC-O_2_: High-Flow Nasal Cannula Oxygen therapy; ISS: Injury Severity Score; NIV: Non—Invasive Ventilation; PaO_2_/FiO_2_ ratio: ratio of arterial oxygen partial pressure to fractional inspired oxygen; PaCO_2_: partial pressure of carbon dioxide; TTSS: Thoracic Trauma Severity Score

Overall, 11 patients (7.8%) required endotracheal intubation for delayed respiratory failure within 2.7 [1.3–5.8] days after randomization (intubation for isolated acute respiratory distress and NIV failure, *N* = 7; intubation for surgery and impossibility of weaning within 8 h postoperatively, *N* = 3; intubation for acute respiratory failure and neurologic impairment [alcohol withdrawal syndrome], *N* = 1).

**Fig. 2:**
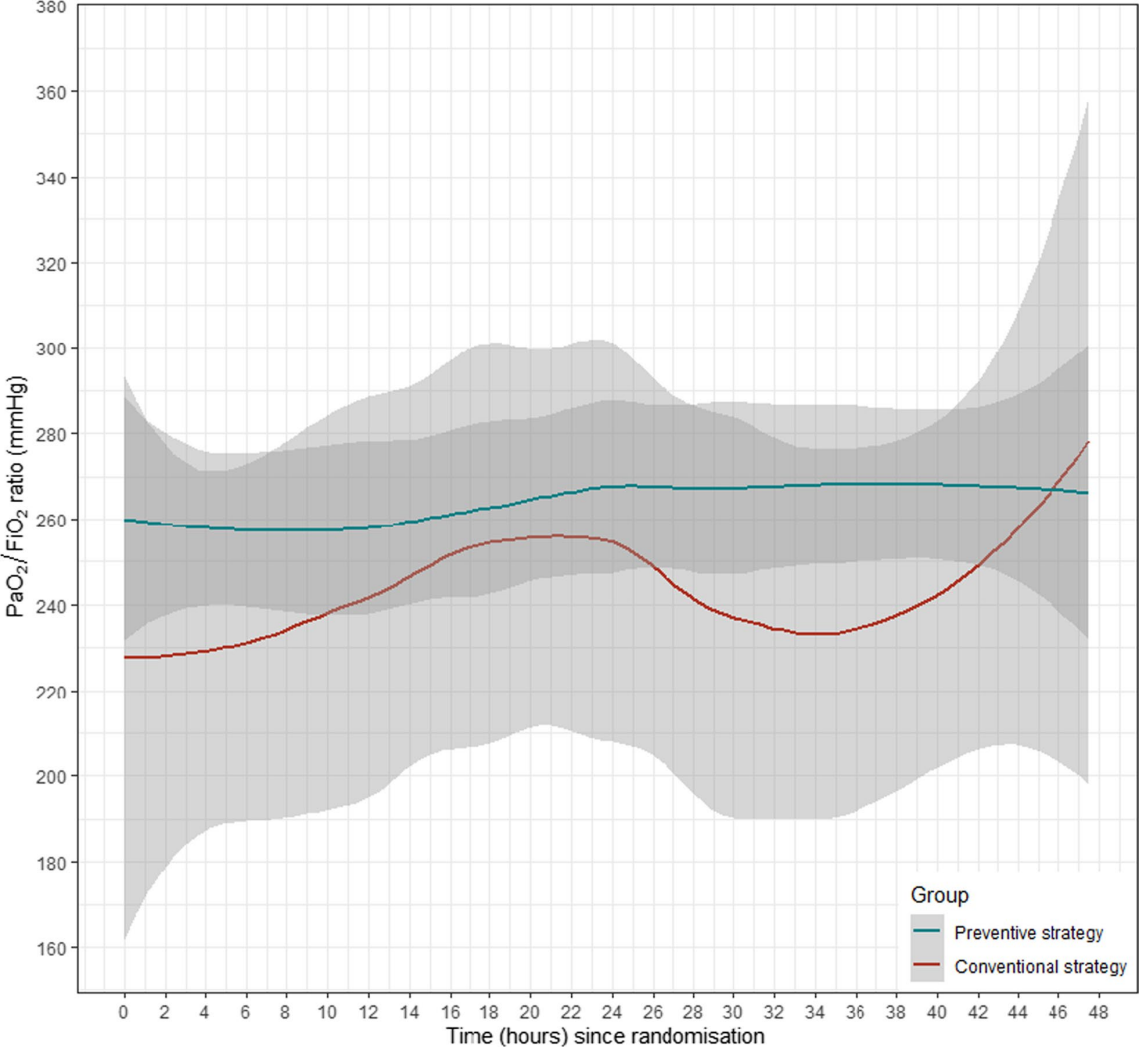

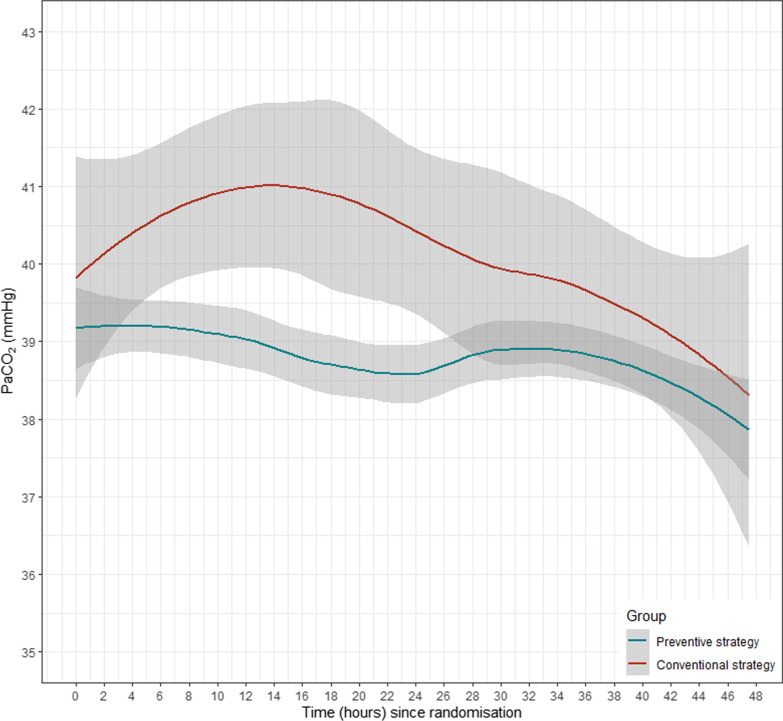
Time course of PaO_2_/FiO_2_ and PaCO_2_ according to the NIV strategy

In this population, the rate of endotracheal intubation was not significantly lower in patients treated with the experimental strategy (7% [5/71]) when compared to the control group (8.6% [6/70]), with an adjusted OR = 0.72 (95%IC: 0.20–2.43), *p* = *0.60*. The time course of PaO_2_/FiO_2_ and PaCO_2_ is shown in Fig. 2, without statistical difference according to the NIV strategy.

The occurrence of pulmonary infection, delayed hemothorax or delayed ARDS was not significantly lower in patients treated by the experimental strategy (adjusted OR = 1.99 [95%IC: 0.73–5.89], *p* = *0.18,* 0.85 [95%IC: 0.33–2.20], *p* = *0.74* and 2.14 [95%IC: 0.36–20.77], *p* = *0.41*, respectively).

Finally, patients allocated to the preventive strategy (HFNC-O_2_ + early NIV) more often experienced excessive agitation with need for sedatives or NIV removal (15 [21%] vs. 2 [3%], with adjusted OR = 12.3 [95%IC: 2.94–112.8], *p* < *0.001*). The rate of other NIV-related side effects was not statistically different between groups, with low rates of secondary pneumothorax or vomiting/aspiration (3 [2%] and 8 [6%], respectively).

## Discussion

Our study failed to demonstrate the interest of a prompt association of HFNC-O_2_ with NIV for preventing endotracheal intubation and secondary respiratory complications in high-risk blunt chest trauma patients with no sign of acute respiratory failure.

Our results are thus in disagreement with a recent meta-analysis supporting the use of non-invasive oxygenation strategies (non-invasive ventilatory support and high-flow nasal oxygen) compared with standard oxygen therapy for preventing endotracheal intubation in patients with mild-to-moderate hypoxemic respiratory failure [16]. However, chest trauma accounted for less than 5% of all-cause respiratory failure, as only few randomized controlled trials suggested the efficacy of NIV or HFNC-O_2_ in this context. Two of these former studies compared NIV to invasive mechanical ventilation [2, 3] and a third only included a very small subgroup of chest trauma patients with acute respiratory failure [4]. To our knowledge, only one randomized controlled trial suggested the efficacy of NIV to prevent intubation in hypoxemic chest trauma patients (defined as having a PaO_2_/FiO_2_ ratio < 200) with no sign of acute respiratory failure [5]. However, several limitations raised concerns about the preventive NIV strategy in this study: (i) patients were severely hypoxemic (mean PaO_2_/FiO_2_ ratio 110 ± 35) and (ii) NIV was performed > 20 h/day over the 48 first hours. In this regard, there is an established evidence-based agreement to avoid delayed intubation in patients with severe hypoxemic respiratory failure [16, 17]. Moreover, an extended use of NIV can raise the concern of inherent complications such as self-inflicted lung injury, gastric distension or secondary pneumothorax [18]. Our results emphasize the poor tolerability of such devices, with higher rates of agitation and claustrophobia, although a recent pilot trial suggested the use of dexmedetomidine to facilitate the acceptance of NIV in chest trauma patients [19, 20].

However, a lack of power may—*at least partially*—explain our negative results, precluding adequate conclusion regarding the most appropriate time for NIV initiation in this context. Several hypothesis can be made to explain an intubation rate lower than expected in the control group (COT ± “late” NIV). First, our sample size calculation relied on former studies reporting a need for mechanical ventilation varying from 17 to 40% in high-risk blunt chest trauma patients with TTSS ≥ 8 or acute respiratory failure [8, 14, 21]. On the other hand, Hernandez et al. reported a lower rate of NIV failure (12%) than those found in former studies where NIV was initiated before the development of respiratory failure [5]. Despite the inclusion of patients with high severity scores, we might thus assume a potential overtriage of patients leading to an overestimated rate of delayed respiratory failure. In agreement with our study, a recent randomized controlled trial did not find any difference between COT and HFNC-O_2_ for prevention of respiratory deterioration (need for unplanned transfer in ICU or escalation of ventilation support) in 220 chest trauma patients at risk for respiratory deterioration [22]. This study was also impaired by an unexpectedly lower incidence of respiratory deterioration than the incidence used for power calculation (6.2% in the HFNP and 6.4% in the COT group). Of note, each of these studies included a bundle of care involving appropriate analgesia and early physiotherapy [5, 22]. In high-risk blunt chest trauma patients, implementation of clinical pathways and multidisciplinary interventions such as effective analgesia, respiratory care and surgical fixation can reduce the rate of secondary respiratory complications [23]. In this regard, the protocolized bundle of care including the early use of locoregional procedure and prompt rehabilitation within the first 48 h after chest trauma may have improved clinical outcome, independently of the use of NIV and HFNC-O_2_.

Several limitations of our study deserve consideration. As mentioned above, there was an unexpected incidence of delayed respiratory failure lower than the incidence used for the power calculation. We thus decided to prematurely stop the trial as the likelihood of finding a treatment effect was unrealistic even if the study was to continue to its full planned sample size, also considering a lower inclusion rate than expected in the context of a worldwide COVID-19 Pandemic. Moreover, the small sample size precluded further analysis of predictors and outcome of NIV failure patients (including ROX or HACOR scale). Finally, the study design and the small subgroup of patients with PaO_2_/FiO_2_ < 200 at enrolment preclude any speculation about the specific effect of HFNC-O_2_ vs. NIV in the most severe patients. Further studies are needed to determine if HFNC-O_2_ is non-inferior to NIV in reducing delayed respiratory failure in blunt chest trauma patients with moderate-to-severe hypoxemia and/or acute respiratory failure [24].

## Conclusion

Early NIV and HFNC-O_2_ compared to COT and late NIV were not shown to be more effective to reduce the need for mechanical ventilation and the rate of secondary respiratory complications in high-risk blunt chest trauma patients with non-severe hypoxemia and no sign of acute respiratory failure. Further adequately powered randomized studies are warranted to provide conclusive evidence.

## Supplementary Information

**Additional file 1:** Study Protocol.

## Data Availability

The datasets used and/or analyzed during the current study are available from the corresponding author on reasonable request.

## Abbreviations

AIS: Abbreviated injury score
ARDS: Acute respiratory distress syndrome
BMI: Body mass index
COT: Conventional oxygen therapy
HFNC-O_2_: High-flow nasal cannula oxygen therapy
ICU: Intensive care unit
ISS: Injury severity score
NIV: Non-invasive ventilation
PaO_2_/FiO_2_ ratio: Ratio of arterial oxygen partial pressure to fractional inspired oxygen
PaCO_2_: Partial pressure of carbon dioxide
TTSS: Thoracic Trauma Severity Score
