# Supplementary material for: Early non-invasive ventilation and high-flow nasal oxygen therapy for preventing endotracheal intubation in hypoxemic blunt chest trauma patients: the OptiTHO randomized trial
Source: Crit Care. 2023 Apr 26;27:163. doi: 10.1186/s13054-023-04429-2 (PMC10131545; doi:10.1186/s13054-023-04429-2)
Supplement: Supplementary file 1 — Additional file 1: Study Protocol. [file 13054_2023_4429_MOESM1_ESM.pdf]

Early non-invasive ventilation and high-flow nasal oxygen therapy for preventing delayed respiratory failure in hypoxemic blunt chest trauma patients. The OptiTHO trial.

Sponsor code: CHUBX 2018/62

INTERVENTIONAL RESEARCH PROTOCOL INVOLVING THE HUMAN PERSON (category 1)

Version n° 1.0 – 18/FEB/2019

N° IDRCB : 2019-A00532-55

Sponsor:

Centre Hospitalier Universitaire de Bordeaux
12, rue Dubernat
33 400 Talence
France

Coordinating investigator:

Matthieu BIAIS, M.D., Ph.D.
Service d'anesthésie réanimation Pellegrin
Centre Hospitalier Universitaire de Bordeaux
Place Amélie Raba Léon
33300 Bordeaux
France

Methodology and Data Management Centre:

Dr Antoine BENARD
Unité de Soutien Méthodologique à la Recherche clinique et épidémiologique
Centre Hospitalier Universitaire de Bordeaux
146 rue Léo Saignat, case n°75 - 33076 Bordeaux Cedex
Tel : 05.57.57.11.29 - Fax : 05.57.57.15.78
Courriel: antoine.benard@u-bordeaux.fr

Clinical research safety and vigilance unit:

Dr Caroline ROUSSILLON
Direction de la recherche clinique et de l'innovation
12, rue Dubernat
33404 Talence Cedex
Tel: 05 57 82 08 34 - Fax: 05 57 82 12 62
vigilance.essais-cliniques@chu-bordeaux.fr

This protocol was designed and written from version 3.0 of 01/02/2017 of the GIRCI SOHO model protocol

HISTORY OF PROTOCOL UPDATES

VERSION	DATE	REASON FOR UPDATE
1.0	18/FEB/2019	

PROTOCOL SIGNATURE PAGE

Sponsor

Centre Hospitalier Universitaire de Bordeaux
12, rue Dubernat
33400 Talence
France

à Bordeaux, le :

Le Directeur du CHU de Bordeaux
P. VIGOUROUX
Et par délégation, le Directeur de la Recherche Clinique et de l'Innovation,
J. BELCASTRO

Coordinating investigator

Matthieu BIAIS, M.D., Ph.D.
Service d'anesthésie réanimation Pellegrin
Centre Hospitalier Universitaire de Bordeaux
Place Amélie Raba Léon
33300 Bordeaux
France
Tel : *05 57 82 10 19*
Fax : *05 57 82 15 06*
Email : matthieu.biais@chu-bordeaux.fr

à Bordeaux, le :

Pr M. BIAIS

Principal Investigator

Cédric CARRIE, M.D.
Service d'Anesthésie Réanimation Pellegrin
Centre Hospitalier Universitaire de Bordeaux
Place Amélie Raba Léon
33300 Bordeaux
France
Tel : 05.56.79.55.95
Fax : 05.56.79.56.72
Email: cedric.carrie@chu-bordeaux.fr

à Bordeaux, le :

Dr C.CARRIE

MAIN CORRESPONDENTS

Coordinating Investigator

Matthieu BIAIS, M.D., Ph.D
Service d'Anesthésie Réanimation Pellegrin
Centre Hospitalier Universitaire de Bordeaux
Place Amélie Raba Léon
33300 Bordeaux
France
Tel : 05 57 82 10 19 - Fax : 05 57 82 15 06
Email : matthieu.biais@chu-bordeaux.fr

Principal Investigator

Cédric CARRIE, M.D.
Service d'Anesthésie Réanimation Pellegrin
Centre Hospitalier Universitaire de Bordeaux
Place Amélie Raba Léon
33300 Bordeaux
France
Tel : 05.56.79.55.95 - **Fax** : 05.56.79.56.72
Email: cedric.carrie@chu-bordeaux.fr

Clinical research safety and vigilance unit

Dr Caroline ROUSSILLON (Head of the Safety and Vigilance Unit), Dr Anne GIMBERT (Pharmacist), Dr Emilie RENARD (Pharmacist), Dr Francesco SALVO (Medical safety manager), Valérie MARTY (Clinical Research Associate), Sandrine VAUTRAT (Clinical Research Associate)
Direction de la recherche clinique et de l'innovation
12, rue Dubernat
33404 Talence Cedex
Phone: 05 57 82 08 34 - Fax: 05 57 82 12 62
vigilance.essais-cliniques@chu-bordeaux.fr

Sponsor

Centre Hospitalier Universitaire de Bordeaux
12 rue Dubernat
33 400 Talence
FRANCE

Sponsor's head of research

Jonathan BELCASTRO
Directeur de la Recherche Clinique et de l'Innovation

Head of Promotion department

Anne GIMBERT
Phone: 05 57 82 08 34 - Fax: 05 56 79 49 26
anne.gimbert@chu-bordeaux.fr

Sponsor's Clinical Research Associate

Sébastien MARCHI
Phone: 05 57 82 04 75-Fax: 05 56 79 49 26
sebastien.marchi@chu-bordeaux.fr

Methodology and Data Management Centre

Unité de Soutien Méthodologique à la Recherche clinique et épidémiologique du CHU de Bordeaux (USMR)
Université Bordeaux Segalen - Case 75
146 rue Léo Saignat - 33076 Bordeaux cedex

Coordinator
Dr. Antoine BENARD
Tél : 05 57 57 11 81 - Fax : 05 57 57 15 78
antoine.benard@u-bordeaux.fr

Statistician
Roxane COUERON
Tél : 05 57 57 56 96 - Fax : 05 57 57 15 78
roxane.coueron@u-bordeaux.fr

Data Manager
Jérôme GALET
Tél : 05 57 57 56 95 - Fax : 05 57 57 15 78
jerome.galet@u-bordeaux.fr

Secreteray
Gwendoline GUNSTER
Tél : 05 57 57 11 29 - Fax : 05 57 57 15 78
gwendoline.gunster@u-bordeaux.fr

CONTENTS

1. SUMMARY OF THE RESEARCH **10**
2. RÉSUMÉ DE LA RECHERCHE **12**
3. SCIENTIFIC JUSTIFICATION AND GENERAL DESCRIPTION **14**
3.1. CURRENT STATE OF KNOWLEDGE 14
3.1.1. Delayed respiratory failure after blunt chest trauma *14*
3.1.2. Non-invasive ventilation in blunt chest trauma : justification and controversies *14*
3.1.3. High-flow nasal oxygen therapy (HFNC-O2) in acute hypoxemic respiratory failure *15*
3.1.4. High-flow nasal oxygen therapy (HFNC-O2) associated to non-invasive ventilation in acute hypoxemic respiratory failure *15*
3.1.5. Research hypotheses and expected results *15*
3.2. JUSTIFICATION OF THE METHODOLOGICAL CHOICES 16
3.2.1. Primary endpoint *16*
3.2.2. Study design *16*
3.2.3. Sub-group analysis *16*
3.3. RISK/ BENEFIT RATIO 16
3.3.1. Individual and collective benefits *16*
3.3.2. Risks and constraints related to the research *16*
4. RESEARCH OBJECTIVES **18**
4.1. MAIN OBJECTIVE 18
4.2. SECONDARY OBJECTIVES 18
5. EVALUATION CRITERIA **18**
5.1. MAIN EVALUATION CRITERION 18
5.2. SECONDARY EVALUATION CRITERION 19
6. RESEARCH DESIGN **19**
6.1. OUTLINE OF THE RESEARCH 19
6.2. METHODS FOR RANDOMISATION 19
7. ELIGIBILITY CRITERIA **20**
7.1. INCLUSION CRITERIA 20
7.2. EXCLUSION CRITERIA 20
7.3. FEASIBILITY AND RECRUITMENT PROCEDURES 20
8. STRATEGIES/ PROCEDURES OF THE RESEARCH **21**
8.1. MANAGEMENT OF NON-INVASIVE VENTILATION 21
8.2. MANAGEMENT OF HIGH-FLOW OXYGEN THERAPY 21
8.3. MANAGEMENT OF OXYGEN THERAPY 21
8.4. EXPERIMENTAL STRATEGY 21
8.5. COMPARISON STRATEGY 21
8.6. SUMMARY FIGURE OF THE PROTOCOL 22
9. ASSOCIATED TREATMENTS AND PROCEDURES **23**
9.1. AUTHORISED ASSOCIATED TREATMENTS AND PROCEDURES 23
9.2. PROHIBITED ASSOCIATED TREATMENTS AND PROCEDURES 23
10. CONDUCTING THE RESEARCH **24**

10.1. THE RESEARCH SCHEDULE 24

10.2. SUMMARY TABLE OF THE PARTICIPANT FOLLOW-UP 24

10.3. PRE-INCLUSION VISIT 24

10.4. INCLUSION VISIT 24

10.4.1. Collection of consent 24
10.4.2. Randomisation procedure 25

10.5. FOLLOW-UP 25

10.6. END OF RESEARCH VISIT 25

10.7. RULES FOR STOPPING THE RESEARCH 25

10.7.1. End of study 25
10.7.2. Discontinuation of research premature 25
10.7.3. Temporary discontinuation of research 26

10.8. PROTOCOL DEVIATIONS 26

10.8.1. Premature and definitive discontinuation of the evaluated procedure 26
10.8.2. Participants wrongly included 26
10.8.3. Patients lost to follow up 26
10.8.4. Other deviations 26

10.9. CONSTRAINTS RELATED TO THE RESEARCH AND POSSIBLE COMPENSATION OF PARTICIPANTS 26

11. MANAGEMENT OF ADVERSE EVENTS AND NEW FACTS 27

11.1. DEFINITIONS 27

11.2. DESCRIPTION OF EXPECTED SERIOUS ADVERSE EVENTS 27

11.3. CONDUCT TO BE MAINTAINED BY THE INVESTIGATOR IN THE EVENT OF AN ADVERSE EVENT, A NEW FACT OR PREGNANCY 28

11.3.1. Collection of adverse events (AE) 28
11.3.2. Notification of serious adverse events (SAEs), adverse events of interest and new facts 28
11.3.3. Notification of pregnancies 28
11.3.4. Summary table of the notification circuit by type of event 29

11.4. DECLARATION BY THE SPONSOR OF UNEXPECTED SERIOUS ADVERSE REACTIONS, NEW FACTS AND OTHER EVENTS 29

11.5. ANNUAL SAFETY REPORT 29

12. STATISTICAL ASPECTS 30

12.1. CALCULATION OF STUDY SIZE 30

12.2. STATISTICAL METHODS EMPLOYED 30

12.2.1. Analysis strategy 30
12.2.2. Descriptive statistical methods 30
12.2.3. Calculation rules in the statistical analyses 30
12.2.4. Software used 31

12.3. ANALYSIS PLAN 31

12.3.1. Description of the inclusions and follow up 31
12.3.2. Patients included in the analysis 31
12.3.3. Baseline characteristics 31
12.3.4. Primary endpoint 31
12.3.5. Secondary endpoint(s) 31

13. MONITORING OF THE RESEARCH 32

13.1. SCIENTIFIC COMMITTEE 32

13.1.1. Composition 32
13.1.2. Frequency of meetings 32
13.1.3. Role 32

13.2. INDEPENDENT DATA SAFETY MONITORING COMMITTEE 33
13.3. ADJUDICATION COMMITTEE 33
13.3.1. Composition 33
13.3.2. Frequency of meetings 33
13.3.3. Role 33
14. RIGHTS OF ACCESS TO DATA AND SOURCE DOCUMENTS 34
14.1. ACCESS TO DATA 34
14.2. SOURCE DATA 34
14.3. DATA CONFIDENTIALITY 34
15. QUALITY CONTROL AND QUALITY ASSURANCE 34
15.1. GUIDELINES FOR COLLECTING DATA 34
15.2. QUALITY CONTROL 34
15.3. DATA MANAGEMENT 35
15.3.1. Software used 35
15.3.2. Data hosting 35
15.3.3. Data security 35
15.3.4. Data entry 35
15.3.5. Access rights 35
15.3.6. Roles 35
15.3.7. Data coding 35
15.3.8. Data testing 35
15.3.9. Reconcile information from the databases AE/SAE 35
15.3.10. Freezing of database 35
15.3.11. Data transfer 36
15.3.12. Archiving the database 36
15.3.13. Quality management 36
15.4. AUDIT AND INSPECTION 36
16. ETHICAL AND REGULATORY CONSIDERATIONS 37
17. PRESERVATION OF RESEARCH DOCUMENTS AND DATA 38
18. FINAL REPORT 38
19. RULES FOR PUBLICATION 38
19.1. SCIENTIFIC COMMUNICATIONS 38
19.2. COMMUNICATION OF RESULTS TO PARTICIPANTS 38
19.3. TRANSFER OF DATA 38
20. BIBLIOGRAPHIC REFERENCES 39

LIST OF ABBREVIATIONS

- AE — Adverse Event
- ANSM — French National Agency for Medicines and Health Products Safety
- COT — Conventional Oxygen Therapy (COT),
- CPP — Committee for the Protection of Persons
- HFNC-O_2 — High-Flow Nasal Cannula Oxygen Therapy
- NIV — Non-invasive ventilation (NIV)
- SAE — Serious Adverse Event
- SUSAR — Suspected Unexpected Serious Adverse Reaction

1. SUMMARY OF THE RESEARCH

**SPONSOR**	Centre Hospitalier Universitaire de Bordeaux
**COORDINATING INVESTIGATOR**	Matthieu BIAIS, M.D., Ph.D.<br>Service d'Anesthésie Réanimation Pellegrin<br>Centre Hospitalier Universitaire de Bordeaux
**TITLE**	Early non-invasive ventilation and high-flow nasal oxygen therapy for preventing delayed respiratory failure in hypoxemic blunt chest trauma patients.<br>The OptiTHO trial
**JUSTIFICATION**	In blunt chest trauma patients without immediate life-threatening conditions, delayed respiratory failure and need for mechanical ventilation may still occur in 12 to 40% of patients, depending on the severity of the trauma, the preexisting conditions and the intensity of initial management.<br>In this context, non-invasive ventilation (NIV) is recommended in hypoxemic chest trauma patients, defined as a PaO_2/FiO_2 ratio < 200 mmHg. However, there is a large heterogeneity among studies regarding the severity of injuries, the degree of hypoxemia and the timing of enrollment. The interest of a preventive strategy during the early phase of blunt chest trauma, before the occurrence of respiratory distress or severe hypoxemia, is not formally established in the literature. Moreover, high-flow nasal oxygen therapy (HFNC-O_2) appears to be a reliable and better tolerated alternative to conventional oxygen therapy (COT), associated with a significant reduction in intubation rate in hypoxemic patients.<br>We hypothesized that an early strategy associating HFNC-O_2 and preventive NIV in hypoxemic blunt chest trauma patients may reduce the need for mechanical ventilation compared to the recommended strategy associating COT and late NIV.
**OBJECTIVES**	The main objective is to compare the rate of endotracheal intubation within 14 days after randomization between two NIV strategies in blunt chest trauma patients:<br>1. A preventive strategy, associating HFNC-O_2 and "early" NIV, in patients having PaO_2/FiO_2 < 300 mmHg (HFNC-O_2 + early NIV)<br>2. The standard of care, associating COT and "late" NIV in patients having PaO_2/FiO_2 < 200 mmHg (COT ± late NIV)
**EVALUATION CRITERIA**	The primary outcome is the proportion of patients who required endotracheal intubation within 14 days after randomization. The following criteria for endotracheal intubation were : cardiac arrest or significant hemodynamic instability, deterioration of neurologic status, signs of persisting or worsening respiratory failure as defined by at least two of the following criteria: respiratory rate of more than 35 breaths per minute, lack of improvement in signs of high respiratory-muscle workload, development of numerous tracheal secretions, signs of respiratory exhaustion (pH <7.32 or $PaCO_2$ > 50 mmHg), severe hypoxemia (PaO_2/FiO_2 ratio < 100 or SpO_2 < 92% for more than 5 minutes).
**OUTLINE OF THE RESEARCH**	Randomized, open-label, multicenter and parallel-group clinical trial comparing two NIV strategies to prevent for delayed respiratory failure in hypoxemic blunt chest trauma patients.
**INCLUSION CRITERIA**	1. Adult patient admitted in intensive care unit within 48 hours after a blunt chest trauma defined by a TTS score ≥ 8<br>2. Presence of an hypoxemia defined by a PaO_2/FiO_2 ratio < 300, and the absence of hypercapnia (PaCO2 ≤ 45 mmHg)<br>3. Without indication of endotracheal intubation at inclusion<br>4. Affiliated person or beneficiary of a social security scheme.<br>5. Free, informed and written consent signed by the participant or next of kin
**EXCLUSION CRITERIA**	1. Exacerbation of underlying chronic respiratory disease, cardiogenic pulmonary edema, severe neutropenia<br>2. Hemodynamic instability, Glasgow score ≤ 12 or excessive agitation, or other contraindications to NIV<br>3. Associated traumatic lesions: severe brain injury, complex facial trauma, tetraplegia, tracheobronchial or esophageal injuries, thoracic or abdominal trauma with indication for surgery by thoracotomy or laparotomy.<br>4. A do-not-intubate order and a decision not to participate.
**RESEARCH TREATMENT/ STRATEGIES/ PROCEDURES**	Two NIV strategies are compared:<br>1. In the experimental strategy, NIV is performed after inclusion in patients with moderate hypoxemia, defined by a PaO_2/FiO_2 ratio < 300 mmHg. The minimally required duration of NIV was 4 hours per day for at least 2 calendar days. The daily duration of NIV can be increased at the discretion of the physician in patients with signs of delayed respiratory failure under HFNC-O_2 and improving under NIV. Beyond the first 48 hours, NIV and HFNC-O_2 can be

	stopped and the patient switched to COT if respiratory rate < 25/min and SpO_2 > 92% under FiO_2 < 30% for at least 6 hours.<br>2. In the control group, patients receive oxygen from nasal cannula or high concentration oxygen mask according to the FiO_2 needed to achieve SpO_2 > 92%. NIV is initiated only in patients having PaO_2/FiO_2 ratio < 200 mmHg under COT. A trial of curative NIV is allowed at the discretion of the physician in patients who have signs of delayed respiratory failure and no other organ dysfunction. The non-improvement of respiratory conditions after 1 hour of NIV, the NIV-dependence (≥ 12 consecutive hours) or NIV-intolerance should be considered as criteria for endotracheal intubation.
**STUDY SIZE**	Hypothesizing that early NIV + HFNC-O_2 may reduce from 25% to 12% the intubation rate, 139 patients per group are required to test this hypothesis, with an 80% power and a 5% risk alpha.<br>Total number of participant expected: 278.
**DURATION OF THE RESEARCH**	− Duration of the inclusion period: 24 months<br>− Participation duration of each participant: 14 days and/or end-of-hospitalization
**STATISTICAL ANALYSIS**	The proportion of patients who required endotracheal intubation within 14 days after randomization will be compared with a Chi² test or with Fisher's exact test, according to the size of the expected values under the hypothesis of independence.<br>Logistic regression model will be used to adjust on stratification factor and other major confounding factors. Assumption of the models (log-linearity of the associations) will be systematically checked. The main analysis will be realized in intention to treat.<br>In a second time, a subgroup analysis, concerning only the patients having PaO2/FiO2 < 200 mmHg at inclusion, will be done to compare the proportion of patients who required endotracheal intubation within 14 days after randomization.
**EXPECTED BENEFITS**	**The expected individual benefits are:**<br>- Preventing endotracheal intubation reduce the specific complications induced by invasive mechanical ventilation (ventilator-associated pneumoniae, ventilation – induced lung or diaphragmatic injury), which is associated with a worse outcome and higher length of stays.<br>- Improving oxygenation and preventing delayed respiratory failure may improve patient comfort and reduce the ICU and hospital length of stay<br>**The expected collective benefits are:**<br>- Establishment of a new preventive strategy including early NIV and HFNC-O_2 in hypoxemic blunt chest trauma patients, incorporated in a bundle of care to reduce the rate of secondary respiratory complications and improve patient outcomes.<br>- Avoiding costs corresponding to the necessary therapies under invasive ventilation (sedation, analgesia or vasopressors), the cost of antimicrobial treatment for infectious complications, the cost of a day under mechanical ventilation, in intensive care and hospital.
**KEY WORDS**	• Chest trauma<br>• Respiratory failure<br>• Noninvasive ventilation<br>• High-flow nasal oxygen therapy

2. RÉSUMÉ DE LA RECHERCHE

PROMOTEUR	Centre Hospitalier Universitaire de Bordeaux
INVESTIGATEUR COORDONNATEUR	Matthieu BIAIS, M.D., Ph.D.<br>Service d'Anesthésie Réanimation Pellegrin<br>Centre Hospitalier Universitaire de Bordeaux
TITRE	Evaluation d'une stratégie préventive associant ventilation non-invasive et oxygénothérapie nasale à haut débit à la phase aiguë d'un traumatisme thoracique hypoxémique. Une étude randomisée.
RATIONNEL	Les traumatismes du thorax sont un motif fréquent d'admission aux urgences et en réanimation. Chez les patients ne présentant pas d'urgence vitale immédiate, le pronostic est étroitement lié à la survenue de complications respiratoires secondaires, survenant dans 12 à 40% des cas selon la gravité des lésions, le terrain du patient et la précocité de la prise en charge.<br>Dans ce contexte, la ventilation non invasive (VNI) est recommandée chez les patients présentant une hypoxémie définie par un rapport PaO2/FiO2 <200 mmHg. Cependant, il existe une très grande hétérogénéité parmi les études concernant la sévérité du traumatisme, le degré d'hypoxémie et le délai d'inclusion. L'intérêt d'une stratégie préventive au cours de la phase précoce d'un traumatisme thoracique, c'est-à-dire avant l'apparition de signes de détresse respiratoire ou d'une hypoxémie sévère, n'est pas formellement établi dans la littérature. De plus, l'oxygénothérapie nasale à haut débit (O2-HDN) semble être une alternative fiable et mieux tolérée que l'oxygénothérapie conventionnelle (OC), associée à une réduction significative du taux d'intubation chez les patients hypoxémiques.<br>Nous avons émis l'hypothèse qu'une stratégie précoce associant HFNC-O2 et VNI préventive chez les patients présentant un traumatisme thoracique hypoxémique pourrait réduire le recours à l'intubation pour détresse respiratoire secondaire par rapport à la stratégie recommandée associant l'OC et la VNI tardive.
OBJECTIFS	L'objectif principal est de comparer deux stratégies différentes pour prévenir le recours à l'intubation chez les patients admis pour traumatisme thoracique hypoxémique :<br>1. Une stratégie précoce, associant VNI + O2-HDN d'emblée chez les patients présentant un rapport PaO2/FiO2 ≤ 300 mmHg.<br>2. La stratégie recommandée, associant OC seule ± VNI curative chez les patients présentant un rapport PaO2/FiO2 ≤ 200 mmHg.
CRITÈRE DE JUGEMENT	Le critère de jugement principal est le recours à une intubation endotrachéale dans les 14 jours suivant la randomisation. Les critères d'intubation endotrachéale sont un arrêt cardiaque ou un état de choc, une altération de l'état neurologique (GCS < 12), des signes de détresse respiratoire définis par au moins deux des critères suivants: fréquence respiratoire (FR) > 35/min, signes de lutte, sécrétions trachéo-bronchiques majeures, signes d'épuisement respiratoire (pH <7,32 ou PaCO2> 50 mmHg) ou hypoxémie majeure (rapport PaO2/FiO2 <100 ou SpO2 <92% pendant plus de 5 minutes).
SCHÉMA D'ÉTUDE	Essai clinique randomisé, ouvert, multicentrique, comparatif sur groupes parallèles portant sur l'évaluation d'une stratégie préventive de ventilation chez les patients traumatisés thoraciques. Stratification par centre et selon le rapport PaO2/FiO2 à l'inclusion.
CRITÈRES D'INCLUSION	1. Patient adulte (> 18 ans) admis en réanimation ou en unité de soins continus dans les 48 heures suivant un traumatisme thoracique à haut risque de complication secondaire (score TTS ≥ 8)<br>2. Présentant une hypoxémie définie par un rapport PaO2/FiO2 ≤ 300, en l'absence d'hypercapnie (PaCO2 ≤ 45 mmHg)<br>3. Sans indication d'intubation endotrachéale lors de la randomisation<br>4. Affilié ou bénéficiaire d'un régime de sécurité sociale<br>5. Signature du consentement éclairé par le patient ou son représentant
CRITÈRES D'EXCLUSION	1. Indication imminente à une intubation endotrachéale (arrêt circulatoire ou respiratoire imminent, état de choc…)<br>2. Patient présentant une contre-indication à la VNI : troubles de la vigilance (score de Glasgow ≤ 12) ou agitation excessive, obstacle des voies aériennes supérieures, syndrome occlusif ou hémorragie digestive haute, état de choc, troubles du rythme ventriculaire grave, sepsis sévère ou choc septique, pneumothorax non drainé (hors pneumothorax minime, unilatéral et sans retentissement clinique), traumatisme facial compliqué de pneumencéphalie, tétraplégie post-traumatique aiguë, plaie trachéo-bronchique, perforation œsophagienne.<br>3. Antécédents pouvant nécessiter une indication de VNI préventive : Comorbidité respiratoire chronique en exacerbation, insuffisance cardiaque congestive, post-opératoire de chirurgie thoracique ou abdominale par thoracotomie ou laparotomie, neutropénie.

	4. Limitation ou abstention thérapeutique, survie ≤ 30 jours<br>5. Critères relatifs à la réglementation : Patient non bénéficiaire d'un régime de sécurité sociale, patient bénéficiant d'une protection renforcée (mineur, femme enceinte ou allaitant), personnes sous protection légale (tutelle ou curatelle), personnes privées de liberté par décision judiciaire ou administrative, personnes séjournant dans un établissement sanitaire ou social, patient inclus dans un autre protocole d'étude biomédicale, patient refusant de donner son consentement.
**DISPOSITIFS / STRATÉGIES / PROCÉDURES**	Deux stratégies d'oxygénation et de ventilation sont comparées<br>1. Une stratégie précoce, associant VNI + O2-HDN d'emblée chez les patients présentant un rapport PaO2/FiO2 ≤ 300 ;<br>2. Une stratégie recommandée, associant OC seule ± VNI curative chez les patients présentant un rapport PaO2/FiO2 ≤ 200.<br>Dans la stratégie précoce (groupe expérimental), la VNI est débutée dès la randomisation chez les patients présentant une hypoxémie modérée, définie par un rapport PaO2/FiO2 ≤ 300. La durée minimale quotidienne de VNI est de 4h par jour sur au moins deux jours consécutifs. La durée peut être augmentée à la discrétion du clinicien chez les patients présentant une dégradation de l'hématose sous O2-HDN et améliorée par VNI. Au-delà des 48 premières heures, la VNI et l'O2-HDN peuvent être sevrés en l'absence de signes de dyspnée hypoxémique (FR < 25 ; SpO2 > 92% sous FiO2 < 30%) pendant au moins 6h.<br>Dans la stratégie recommandée (groupe contrôle), les patients sont traités par OC via une canule nasale ou un masque à oxygène dont le débit est adapté pour un objectif de SpO2 > 92%. La VNI n'est initiée que chez les patients ayant un rapport PaO2/FiO2 ≤ 200 mmHg sous OC. Une séance de VNI curative est autorisée à la discrétion du clinicien chez les patients présentant des signes d'insuffisance respiratoire sans autre défaillance d'organe. La non-amélioration des paramètres respiratoires après 1 heure de VNI et/ou une dépendance à la VNI (≥ 12 heures consécutives) et/ou une intolérance à la VNI doivent être considérés comme des critères d'intubation endotrachéale.
**NOMBRE DE PATIENTS**	L'hypothèse de cette étude est une réduction du taux d'intubation de 25% dans le groupe contrôle à moins de 12% dans le groupe expérimental. 139 patients par groupe sont nécessaires pour tester cette hypothèse avec une puissance de 80% et un risque $\alpha < 5\%$.<br>Nombre total de sujets à inclure : 278.
**DURÉE DE LA RECHERCHE**	Durée des inclusions: 24 mois<br>Durée de participation pour chaque participant : 14 jours et/ou fin d'hospitalisation
**ANALYSE STATISTIQUE**	Le taux de patients ayant recours à une intubation endotrachéale dans les 14 jours suivant la randomisation sera comparée par un test du Chi² ou un test exact de Fisher. Un modèle de régression logistique sera utilisé pour ajuster selon les facteurs de stratification et d'autres facteurs de confusion majeurs. L'analyse principale sera réalisée en intention de traiter. Dans un deuxième temps, une analyse de sous-groupe, portant uniquement sur les patients ayant une PaO2 / FiO2 <200 mmHg à l'inclusion, sera effectuée afin de comparer la proportion de patients nécessitant une intubation endotrachéale dans les 14 jours suivant la randomisation.
**RETOMBÉES ATTENDUES**	**Les bénéfices individuels attendus sont:**<br>- La prévention de l'intubation endotrachéale, la réduction des complications induites par la ventilation mécanique invasive (pneumopathies acquises sous ventilation, lésions pulmonaires ou diaphragmatiques induites par la ventilation).<br>- L'amélioration de l'oxygénation et l'amélioration du confort du patient.<br>- La réduction des durées de séjour.<br>**Les bénéfices collectifs attendus sont:**<br>- La démonstration de l'efficacité d'une stratégie préventive intégrée dans une prise en charge globale et précoce du traumatisme thoracique, pouvant à terme modifier l'orientation des patients et les recommandations de prise en charge.<br>- Eviter les coûts correspondant aux traitements nécessaires sous ventilation invasive (sédation, analgésie ou vasopresseurs), le coût du traitement antimicrobien pour complications infectieuses, le coût d'une journée en ventilation mécanique, en réanimation et en hôpital.
**MOTS CLÉS**	Traumatisme thoracique<br>Détresse respiratoire<br>Ventilation non-invasive<br>Oxygénothérapie nasale à haut débit

3. SCIENTIFIC JUSTIFICATION AND GENERAL DESCRIPTION

3.1. CURRENT STATE OF KNOWLEDGE

3.1.1. DELAYED RESPIRATORY FAILURE AFTER BLUNT CHEST TRAUMA

Blunt chest trauma frequently involves multiple rib fractures resulting in a painful limitation of thoracic amplitude and a reduction of coughing. The parietal restrictive syndrome is exacerbated by the reduction in functional residual capacity due to pleural effusion or alveolar contusion. This triggers a vicious circle with bronchial congestion, alveolar consolidation and atelectasis, leading to delayed respiratory failure, need for prolonged ventilation and possible death [1].

In high-risk blunt chest trauma patients, multidisciplinary interventions such as effective analgesia, respiratory care and surgical fixation may reduce the rate of delayed respiratory failure, prevent the use for mechanical ventilation and improve patient outcomes [2]. Moreover, an early management through appropriate orientation seems paramount in order to prevent under-triage, associated with increased use of health-care resources and long-term disabilities [3].

On the other hand, previous research highlighted the difficulties in identifying high-risk trauma patients without immediate life-threatening injuries [3]. The TTS score (Thorax Trauma Severity Score) seems the more suitable for prediction of post-traumatic complications and outcome in blunt chest trauma (**Table 1**) [4].

Delayed respiratory failure and need for mechanical ventilation may still occur in 12 to 40% of patients, depending on the severity of the trauma, the preexisting conditions and the intensity of initial management [5]. Mechanical ventilation and prolonged weaning can lead to specific complications such as ventilator-acquired pneumonia [6], ventilation-induced lung or diaphragmatic injuries [7,8] and are associated with an increase in morbidity, mortality, length of stay and costs [9].

Table 1. Thoracic Trauma Severity Score, *adapted from [3, 4]*

PaO_2/FiO_2	Rib fracture	Contusion	Pleural involvement	Age (years)	Points
>400	0	None	None	<30	0
300–400	1-3	1 lobe	Pneumothorax	30–41	1
200–300	4–6 unilateral	1 lobe bilateral or 2 lobes unilateral	Unilateral HT or HPT	42–54	2
150–200	>3 bilateral	<2 lobes bilateral	HT or HPT bilateral	55–70	3
<150	Flail chest	≥2 lobes bilateral	Tension pneumothorax	>70	5

All categories have to be added to achieve a score ranging from 0 to 25. HT, haemothorax; HPT, hemopneumothorax.

3.1.2. NON-INVASIVE VENTILATION IN BLUNT CHEST TRAUMA : JUSTIFICATION AND CONTROVERSIES

The rational supporting the use of NIV in chest trauma patients is supported by the meta-analysis of Chiumello et al. [10] that included 10 studies (4 randomized trials and 6 observational studies) and suggested an association between the use of non-invasive ventilation and a decrease in mortality (OR = 0.26 [0.09-0.71]), the need for intubation (RR = 0.32 [0.12 - 0.86]) and the incidence of nosocomial pneumonia (RR = 0.34 [0.2 - 0.58]).

However, only two randomized trials compared NIV to conventional oxygen therapy (COT) [11, 12], the other compared NIV to mechanical invasive ventilation [13, 14]. In addition, one randomized trial assessed the value of NIV in hypoxemic acute respiratory failure and included only a minority of thoracic traumatized patients (and a majority of cardiogenic pulmonary edema) [11]. Other meta-analysis excluding the study of Ferrer et al. didn't find any beneficial effect of NIV in mortality [15, 16].

In most studies, NIV was instituted when patients had already developed acute respiratory decompensation associated with hypoxemia. To our knowledge, only one randomized controlled trial of moderate quality suggested the efficacy of NIV to prevent intubation in hypoxemic chest trauma patients [12], but several limitations raised concerns about the preventive strategy of NIV in this study: (1) patients were severely hypoxemic (mean PaO_2/FiO_2 ratio 100±35), (2) NIV was performed > 20h/day and (3) indications for intubation were not defined. Controversially, delaying intubation in NIV-depending patients may be detrimental [17].

Given the positive overall results, the cautious use of non-invasive ventilation (NIV) is recommended to prevent intubation in hypoxemic chest trauma patients, defined by a PaO2/FiO2 ratio < 200 [18]. Data reported by Hernandez et al. suggested that early identification of at-risk patients with prompt institution of preventive NIV in appropriate patients may be of greatest benefit because NIV failure and mortality rates were lower than those found in the studies where NIV was initiated following the development of respiratory failure [16]. The heterogeneity in the trials design, the severity of the patients at inclusion or the difference in comparators (i.e. oxygen or invasive ventilation) however precludes definitive conclusion regarding the most appropriate time for the introduction of NIV in this context [19].

3.1.3. High-flow nasal oxygen therapy (HFNC-O2) in acute hypoxemic respiratory failure

High-flow nasal oxygen therapy (HFNC-O_2) exerts multiple physiologic effects leading to a significant improvement of oxygenation, patient comfort and work of breathing compared to conventional oxygen therapy (COT) in patients with acute hypoxemic respiratory failure [20-22]. To our knowledge, no study has however evaluated HFNC-O_2 in blunt chest trauma patients.

The rational of HFNC-O_2 in this context is first supported by the randomized trial of Stéphan et al. that demonstrated the non-inferiority of HFNC-O_2 compared to NIV for the prevention or treatment of respiratory distress in 830 postoperative cardio-thoracic surgery patients [23]. The reintubation rate was similar in both groups (21 vs. 22%), with no difference in mortality. Skin lesions were more common in the NIV group.

Moreover, the multicenter randomized trial of Frat et al. compared HFNC-O_2 with NIV and COT in 310 critically ill patients admitted for an acute hypoxemic respiratory distress [24]. The inclusion criteria were: respiratory rate> 25/min, PaO_2/FiO_2 ratio ≤ 300 under oxygen therapy> 10L /min for at least 15min, PaCO2 ≤ 45mmHg. Patients with decompensated obstructive pulmonary comorbidity, cardiogenic pulmonary edema, febrile neutropenia or contraindication to NIV were excluded. The rates of intubation were not statistically different between HFNC-O_2 (38% [40/106]), NIV (50% [55/110]) and COT (47% [44/94]), but the number of ventilator-free days at day 28 was significantly higher and the hazard ratio for death at 90 days was significantly lower in the HFNC-O_2 group.

3.1.4. High-flow nasal oxygen therapy (HFNC-O2) associated to non-invasive ventilation in acute hypoxemic respiratory failure

Several points justify this strategy:

1. The use of NIV is recommended by international guidelines for preventing intubation in hypoxemic blunt chest trauma patients [18, 19]. The interest of a preventive strategy during the early phase of blunt chest trauma, before the occurrence of respiratory distress or severe hypoxemia, is not formally established by the literature.
2. Oxygenation strategies with HFNC-O_2, alone or in combination with NIV, have not been studied in chest trauma patients. A third group associating curative early HFNC-O_2 ± late VNI would have required a non-inferiority hypothesis between preventive VNI + HFNC-O_2 strategy and HFNC-O_2 ± late VNI.

3.1.5. Research hypotheses and expected results

The **best ventilation strategy** to prevent delayed respiratory failure and need for mechanical ventilation remains to be determined. To our knowledge, no study has compared two NIV strategies during the management of hypoxemic blunt chest trauma patients:

1. The standard of care, associating COT and "late" NIV, in patients having PaO_2/FiO_2 ratio < 200 mmHg (COT ± late NIV)

2. A preventive strategy, associating HFNC-O_2 and NIV during the early management of trauma patients having PaO_2/FiO_2 ratio < 300 mmHg (HFNC-O_2 + early NIV)

We hypothesized that an early strategy associating HFNC-O_2 and preventive NIV in hypoxemic blunt chest trauma patients may reduce the need for mechanical ventilation compared to the recommended strategy associating COT and late NIV, without increasing ICU or hospital length of stays.

3.2. JUSTIFICATION OF THE METHODOLOGICAL CHOICES

3.2.1. PRIMARY ENDPOINT

Our primary endpoint is **the necessity to perform endotracheal intubation within 14 days after randomization**. This endpoint is extensively used in the literature [11, 12, 24].
The 14-day period after randomization is justified by the following arguments:

1. Secondary respiratory complications after blunt chest trauma occur during the first week, mainly between 3 to 5 days [1, 6, 31].
2. The median ICU length of stay of a thoracic traumatism in our institution is 6 [4 - 8] days [31].
3. The study of Frat et al. found no significant increase in the intubation rate after 12 days following acute hypoxemic respiratory distress [24].

The extension of the follow-up time may overestimate the rate of intubation for non-traumatic related causes (secondary complications related to hospitalization)

3.2.2. STUDY DESIGN

We will conduct an open-label, parallel-groups, randomized clinical trial of superiority. One of the potential biases in this intensive-care trial is that the intensivist behavior might be influenced by the result of the randomization, particularly in his decision to perform endotracheal intubation or not. The risk is to underestimate the need for endotracheal intubation in the preventive strategy (HFNC-O2 and early NIV).

To limit the possibility of inducing this measurement bias, we will:

1. Clearly define the criteria for endotracheal intubation. See chapter 4.1 for details.
2. Designate an adjudication committee that will validate all endotracheal intubation, based on clinical and biological data, blinded from the strategy used.

3.2.3. SUB-GROUP ANALYSIS

A two parallel groups design will not allow estimating the specific effect of HFNC-O2 compared to COT with or without NIV. A subgroup analysis may allow estimating the specific effect of HFNC-O2 in patients with severe hypoxemia at inclusion (PaO2/FiO2 ratio < 200 mmHg). Indeed, all patients with severe hypoxemia at inclusion will be treated by NIV, associated with COT in the standard of care and with HFNC-O2 in the preventive strategy. Randomization will be stratified on PaO2/FiO2 ratio at inclusion to allow this subgroup analysis.

3.3. RISK/ BENEFIT RATIO

3.3.1. INDIVIDUAL AND COLLECTIVE BENEFITS

Prompt institution of preventive NIV is expected to prevent delayed respiratory failure, NIV failure and need for mechanical ventilation [10, 16]. Consequently, preventive NIV is thought to decrease specific complications of mechanical ventilation, such as ventilator-acquired pneumonia or prolonged weaning [6]. Thus, the ICU and hospital length of stay should not be longer despite the increased level of care at the early phase.

Moreover, HFNC-O2 should improve oxygenation and patient comfort when compared to COT. The prompt association with preventive NIV should be associated with a significant reduction in NIV dependency, NIV failure and need for mechanical ventilation.

The expected collective benefits are:

- Early identification of high-risk patients to prevent under-triage, associated with increased use of health-care resources and long-term disabilities [3].
- Establishment of a new preventive strategy including HFNC-O2 associated with non-invasive ventilation in hypoxemic blunt chest trauma patients.
- Avoided costs corresponding to the necessary treatments under invasive mechanical ventilation (hypnotic, analgesic or even vasopressor), the cost ventilator-associated pneumonia and the cost of a day under mechanical ventilation in intensive care unit...

3.3.2. RISKS AND CONSTRAINTS RELATED TO THE RESEARCH

When considering a trial of NIV in patients with blunt chest trauma, the most important individual risks are closely related to the indications for endotracheal intubation. For patients who are unresponsive to NIV,

endotracheal intubation should be considered early to mitigate the potential for harm. Delaying intubation in NIV-depending patients may be detrimental [25-28]. In this context, Carrillo et al. previously demonstrated that duration of NIV before intubation was significantly associated with decreased survival rate (OR adjusted 0.98 [0.96 - 0.99]) [30].

In order to minimize the risks associated with a “too early” or “too late” intubation, criteria for delayed respiratory failure needing mechanical ventilation are given in the study protocol (4.1 main judgment criteria).

Moreover, the use of NIV in blunt chest trauma may present inherent risks regardless of the specific contraindications [29, 30]. The adverse effects reported in the literature are (in order of frequency): facial mask-related discomfort (30-50%), gastric distension (30-40%), cutaneous lesions (10-20%), claustrophobia or anxiety (10-20%), inhalation (5%) and pneumothorax (rare). NIV contraindications are a non-inclusion criterion for the present study.

In order to minimize the adverse effects related to NIV, NIV should be initiated 4 hours a day for 48h, intensified only according to the clinical course of the patient (**5.2 METHODS OF ADMINISTRATION OF NIV**). Tolerance of NIV will be assessed during the course of the study and may lead to a cessation of the procedure.

Other constraints are related to the need of additional biological examinations, particularly arterial gas sampling. Patients discomfort is limited by the introduction of an arterial catheter.

4. RESEARCH OBJECTIVES

4.1. MAIN OBJECTIVE

The main objective of this study is to compare the rate of endotracheal intubation within 14 days after randomization between two NIV strategies in blunt chest trauma patients:

1. **An "early" NIV strategy associated with HFNC-O_2 (experimental group)**
2. **A "late" NIV strategy associated with COT (control group)**

4.2. SECONDARY OBJECTIVES

To compare the rate of endotracheal intubation within 14 days after randomization between an "early" NIV strategy associated with HFNC-O2, and a "late" NIV strategy associated with COT, in blunt chest trauma patients with PaO_2/FiO_2 ratio < 200 mmHg at inclusion.

The other secondary objectives are to compare between the two strategies:

- The evolution of the PaO_2/FiO_2 ratio during the first 48 hours,
- The evolution of the respiratory rate during the first 48 hours,
- The evolution of dyspnea score during the first 48 hours
- The total duration of NIV, the total duration of invasive ventilation and the number of days without ventilation (invasive and non-invasive) between admission and Day 14
- The occurrence of secondary complications (severe hypoxemia [PaO_2/FiO_2 ratio < 200], nosocomial pneumonia or bacteremia, septic shock, transfusion of blood product, secondary pleural effusion with chest tube requirement) between admission and Day 14
- The length of stay in intensive care unit and in hospital
- The mortality rate in intensive care unit and in hospital

5. EVALUATION CRITERIA

5.1. MAIN EVALUATION CRITERION

The main evaluation criterion is the necessity to perform endotracheal intubation within 14 days after randomization.

To ensure the consistency of indications across sites and reduce the risk of delayed intubation, the following criteria for endotracheal intubation must be used (only one criterion is needed) [24]: cardiac arrest or significant hemodynamic instability, deterioration of neurologic status, signs of persisting or worsening respiratory failure as defined by at least two of the following criteria: respiratory rate of more than 35 breaths per minute, lack of improvement in signs of high respiratory-muscle workload, development of copious tracheal secretions, signs of respiratory exhaustion (pH <7.32 or $PaCO_2$ > 50 mmHg), major hypoxemia (PaO_2/FiO_2 ratio <100 or SpO_2 <92% for more than 5 minutes).

A trial of curative NIV is allowed at the discretion of the physician in patients who have signs of persisting or worsening respiratory failure and no other organ dysfunction. The non-improvement of respiratory conditions after 1 hour of NIV, the NIV-dependence (≥ 12 consecutive hours) or NIV-intolerance should be considered as criteria for endotracheal intubation.

For patients requiring urgent or scheduled surgery after randomization, endotracheal intubation for general anesthesia is not considered a failure of the NIV strategy, provided that the patient can be weaned from the mechanical ventilation within 8 hours postoperatively. The reason for mechanical ventilation should be recorded for each patient (acute respiratory failure, other complication, general anesthesia for scheduled surgery).

5.2. Secondary evaluation criterion

The PaO2/FiO2 ratio, the respiratory rate and the dyspnea score (*see below*) will be measured every 6 hours during the first 48 hours.

**Dyspnea score**
+2: significant improvement
+1: slight improvement
0: no change
-1: slight deterioration
-2: significant deterioration

Secondary outcomes were the ICU and hospital length of stay, the ICU or in-hospital mortality, the number of ventilator free-days (i.e., days alive and without invasive or non-invasive mechanical ventilation) between day 1 and day 14.

The occurrence of secondary respiratory complications such as the incidence and duration of severe hypoxemia (defined by a PaO_2/FiO_2 ratio < 200), the rate of nosocomial pneumonia or secondary pleural effusion needing chest tube insertion, was also recorded. Health-care associated pneumonia and/or ventilator acquired pneumonia was defined by a new pulmonary infiltrates in plain chest radiograph, persistent fever or hypothermia, leukopenia or leukocytosis and at least two of the following criteria: cough or dyspnea, purulent bronchial secretions, oxygen requirement [33]. Positivity thresholds for quantitative culture results were ≥ 10^4 UFC/mL for BALs, ≥ 10^6 UFC/mL for endotracheal aspirations and ≥ 10^7 for non-invasive sputum samples [34].

Other prespecified outcomes included other complications during the ICU stay, such as septic shock, cardiac arrest or severe arrhythmia, vasopressor requirement, transfusion of blood product. Finally, adverse effects of NIV or HFNC-O_2 were recorded: gastrointestinal intolerance (abdominal distension, vomiting and inhalation), skin lesions, conjunctival irritation...

6. RESEARCH DESIGN

6.1. Outline of the research

This is a randomized, open-label, multicenter and parallel-group clinical trial comparing two NIV strategies to prevent for delayed respiratory failure in hypoxemic blunt chest trauma patients:

- An early NIV strategy associated with HFNC-O2 (experimental group)
- A late NIV strategy associated with COT (control group)

This study didn't aim to evaluate devices (NIV or HFNC-O_2) which are used routinely in intensive care settings.

6.2. Methods for Randomisation

The randomization list is established by the statistician of the Methodology and Data Management Centre before the start of the trial. The numbers of 2 randomized groups are balanced with ratio 1:1. A document describing the randomization procedure will be kept in a confidential and secure place in the Methodology and Data Management Centre.

Randomization of patients is performed the day of the inclusion after verifying the eligibility of the participant, using a secured centralized e-CRF. Information on the randomization group for the patient is given by the e-CRF to the study investigator.

Randomization in 2 groups with stratification by center and by PaO_2/FiO_2 values (< or ≥ 200 mmHg):

- Group 1: HFNC-O2 + "early" NIV in patients having PaO2/FiO2 < 300 mmHg
- Group 2: COT ± "late" NIV in patients having PaO2/FiO2 < 200 mmHg

Practical procedure to randomize a patient is described in chapter 9.4 of this protocol.

7. ELIGIBILITY CRITERIA

7.1. INCLUSION CRITERIA

All consecutive patients who are 18 years of age or older may be enrolled if they meet all of the following criteria:

1. Patient admitted in intensive care unit within 48 hours after a high-risk blunt chest trauma, defined by a TTS score ≥ 8 (**Table 1**).
2. Hypoxemia defined by a PaO_2/FiO_2 ratio < 300, and the absence of hypercapnia ($PaCO_2$ < 45 mmHg).
3. Without indication of endotracheal intubation at inclusion.
4. Affiliated person or beneficiary of a social security scheme.
5. Free, informed and written consent signed by the participant and the investigator (at the latest on the day of inclusion and before any examination required by the research).

7.2. EXCLUSION CRITERIA

The non-inclusion criteria are:

1. Criteria relating to formal indication to NIV: Exacerbation of underlying chronic respiratory disease, cardiogenic pulmonary edema, severe neutropenia.
2. Criteria relating to contraindications to NIV: Hemodynamic instability, Glasgow Coma Scale score ≤ 12 or excessive agitation, or other contraindications to non-invasive ventilation (active gastrointestinal bleeding, low level of consciousness, multiorgan failure, airway patency problems, lack of cooperation or hemodynamic instability).
3. Associated traumatic lesions entailing particular risks: severe brain injury, complex facial trauma, tetraplegia, tracheobronchial or esophageal injuries, thoracic or abdominal trauma with indication for surgery by thoracotomy or laparotomy.
4. Criteria relating to the regulation: A do-not-intubate order and a decision not to participate, persons placed under judicial protection, persons participating in another research including a period of exclusion still in course, severely altered physical and/or psychological health which, according to the investigator, could affect the participant's compliance of the study.

7.3. FEASIBILITY AND RECRUITMENT PROCEDURES

Recruitment and inclusion can be performed either in the ICU or in the emergency settings, if equipped with NIV and HFNC-O_2 devices. As previously described in former studies, the 48-hr delay between the initial trauma and inclusion allow the primary transfer in a Level 1 or 2 trauma center, the stabilization of the patient and the emergency management of traumatic injuries and the risk-assessment indicating secondary transfer in intensive care settings [12].

The main investigator center can demonstrate an inclusion capacity > 30 patients/year, testified by a pilot study and participation to a multicenter trial evaluating the medico-economic interest of an early surgical management for rib fractures in severe chest trauma [33].

Fifteen recruiting centers have been selected to participate in the study. All of them are Level 1 or 2 Trauma Centers, with a great expertise in management of thoracic trauma according to the most recent recommendations. In feasibility survey, centers have witnessed an inclusion capacity > 10 patients / year (1 patient/month/center).

The anticipated duration of recruitment is 24 months.

8. STRATEGIES/ PROCEDURES OF THE RESEARCH

Two NIV strategies are compared in hypoxemic blunt chest trauma patients:

1. A preventive strategy, associating HFNC-O_2 and NIV during the early management of trauma patients with PaO_2/FiO_2 ratio < 300 mmHg (HFNC-O_2 + early NIV = experimental strategy)

2. The standard of care, associating COT and "late" NIV, in patients with severe hypoxemia or respiratory failure (COT ± late NIV = comparison strategy)

This study doesn't aim to evaluate devices (NIV or HFNC-O2) which are used routinely in intensive care settings.

8.1. Management of non-invasive ventilation

Non-invasive ventilation is delivered to the patient through a face mask connected to an ICU ventilator, with pressure support applied in a NIV mode. All conventional measures must be applied to minimize leakage. The pressure-support level is initially set at 0 cmH_2O (CPAP) and then increased by steps of 2 cmH_2O with the aim of obtaining an expired tidal volume of 7 to 10 ml/kg of predicted body weight with a respiratory rate <25/min. The initial FiO_2 is adjusted to maintain a SpO_2 > 92% or PaO_2 > 65 mmHg. The level of positive expiratory pressure is initially set at 5 cmH_2O and then incrementally increased by 1 cmH_2O to obtain SpO_2 > 92% or PaO_2 > 65 mmHg with minimal FiO_2, while minimizing patients' discomfort and leaks around the mask.

8.2. Management of high-flow oxygen therapy

In the high-flow oxygen group, oxygen was passed through a heated humidifier (MR850, Fisher and Paykel Healthcare) and applied continuously through large-bore binasal prongs (Optiflow, Fisher and Paykel Healthcare). The fraction of oxygen in the gas flowing in the system is adjusted to maintain a SpO2 > 92% or PaO2 > 65 mmHg. The gas flow rate between 30 and 70 L/min is subsequently adjusted as follows according to the tolerance of the patient.

**FiO2**	21 - 30%	30 - 40%	40 - 60%	60 - 100%
**Gas Flow Rate**	30 L/min	30 - 40L/min	40 - 50 L/min	50 - 70 L/min

8.3. Management of oxygen therapy

Oxygen therapy is administered from nasal cannula or high concentration oxygen mask according to the FiO2 needed to achieve SpO2 > 92%.

8.4. Experimental strategy

In the experimental strategy, NIV is performed just after inclusion of patients having moderate hypoxemia, defined by a PaO_2/FiO_2 ratio < 300 mmHg.

The minimally required duration of noninvasive ventilation was 4 hours per day for at least 2 calendar days. Between NIV sessions, high-flow oxygen must be applied for at least 2 calendar days after inclusion.

The daily duration of NIV can be increased at the discretion of the physician in patients who have signs of delayed respiratory failure under HFNC-O_2 and improving under NIV. The non-improvement of respiratory conditions after 1 hour of NIV, the NIV-dependence (≥ 12 consecutive hours) or NIV-intolerance should be considered as criteria for endotracheal intubation (see 4.1 MAIN EVALUATION CRITERION).

Beyond the first 48 hours, HFNC-O_2 and NIV can be stopped and the patient switched to standard oxygen therapy if respiratory rate < 25/min and SpO_2 > 92% under FiO_2 < 30% for at least 6 hours.

8.5. Comparison strategy

In this group, NIV should only be initiated:

- In severely hypoxemic patients, defined by a PaO_2/FiO_2 ratio < 200 mmHg under COT.

- In patients who have signs of delayed respiratory failure and no other organ dysfunction (see **4.1 MAIN EVALUATION CRITERION**).

The non-improvement of respiratory conditions after 1 hour of NIV, the NIV-dependence (≥ 12 consecutive hours) or NIV-intolerance should be considered as criteria for endotracheal intubation (see **4.1 MAIN EVALUATION CRITERION**).

8.6. SUMMARY FIGURE OF THE PROTOCOL

All consecutive patients may be enrolled if they meet <u>all of the following criteria</u>:

- Adult patient admitted within 48 hours after a high-risk blunt chest trauma, defined by a TTS score ≥ 8
- Having an hypoxemia defined by a PaO_2/FiO_2 ratio ≤ 300, in the absence of hypercapnia ($PaCO_2$ ≤ 45 mmHg)
- Without indication of endotracheal intubation at inclusion
- Affiliated person or beneficiary of a social security scheme
- Free, informed and written consent signed by the participant and/or next of kin and the investigator

Without any exclusion criteria:

- Exacerbation of underlying chronic respiratory disease, cardiogenic pulmonary edema, severe neutropenia
- Hemodynamic instability, Glasgow score ≤ 12 or excessive agitation, or other contraindications to NIV
- Severe brain injury, complex facial trauma, tetraplegia, tracheobronchial or esophageal injuries, surgery by thoracotomy or laparotomy
- A do-not-intubate order, judicial protection, severely altered physical and/or psychological health.

EXPERIMENTAL STRATEGY
$HFNC-O_2$ + early NIV

COMPARATIVE STRATEGY
COT ± late NIV

During the first 2 days
- **VNI** ≥ 4h/day
- **$HFNC-O_2$** QSP SpO_2 > 92%

PaO_2/FiO_2 ≤ 200
&/or acute respiratory failure
COT + NIV

200 < PaO_2/FiO_2 ≤ 300
Without acute respiratory failure
COT

Worsening of patient's conditions:
↗ NIV if no criteria for intubation

Worsening of patient's conditions
COT + NIV

Improvement of patient's conditions:
↕ NIV ± $HFNC-O_2$ if RR < 25/min and SpO_2 > 92% under FiO_2 < 30%

Criteria for endotracheal intubation (only one is needed): cardiac arrest or significant hemodynamic instability, deterioration of neurologic status, signs of persisting or worsening respiratory failure: respiratory rate > 35 / min, signs of high respiratory-muscle workload, development of copious tracheal secretions, signs of respiratory exhaustion (pH <7.32 or $PaCO_2$ > 50 mmHg), major hypoxemia (PaO_2/FiO_2 ratio <100 or SpO_2 <92% for more than 5 minutes).
A trial of curative NIV is allowed in patients who have signs of persisting or worsening respiratory failure and no other organ dysfunction. The non-improvement of respiratory conditions after 1 hour of NIV, the NIV-dependence (≥ 12 consecutive hours) or NIV-intolerance should be considered as criteria for endotracheal intubation.

9. ASSOCIATED TREATMENTS AND PROCEDURES

9.1. Authorised associated treatments and procedures

In high-risk blunt chest trauma patients, multidisciplinary interventions such as effective analgesia, respiratory care and eventually surgical fixation may reduce the rate of delayed respiratory failure, prevent the use for mechanical ventilation and improve patient outcomes.

The bundle of care for blunt chest trauma, adapted from the up-to-date recommendations [18, 33], is resumed as followed:

**1. Pain control**
**1.1. Repeated assessment of visual analog pain scale (VAS)**
At rest, coughing and deep breathing
Goals of pain relief : VAS ≤ 4 both at rest and during physical effort (i.e. cough and deep breathing)
**1.2. Intravenous analgesia**
Morphine and/or ketamine titration until resting VAS < 3.
Addition of morphine intravenous patient-controlled analgesia if persistence of VAS > 3.
Use of intravenous co-analgesics as a multimodal opioid-saving strategy, including non-steroid anti-inflammatory drugs (NSAIDs) unless absolute contra-indication.
**1.3. Locoregional analgesia**
Within 12 hours after randomization if persistence of a VAS > 4 and / or FVC < 50% despite optimized intravenous analgesia
Indication and technique (epidural or paravertebral catheter, Erector Spinae Plane Block or Serratus Plane Block) left to the discretion of the attending physician unless specific contra-indication
**2. Chest tube insertion**
If complete pneumothorax, and in case of any liquid or air effusion that leads to respiratory and/or hemodynamic consequences
If hemothorax estimated > 500ml (ultrasound or CT scan evaluation)
Indication and technique left to the discretion of the attending physician. Pigtail catheters preferred in isolated pneumothorax with low risk of secondary hemothorax.
In case of minor pneumothorax and without clinical consequences, drainage is not systematic. In these situations, NIV is not contraindicated provided clinical observation and repeated chest X-ray.
**3. Surgical advice**
Discuss indication of osteosynthesis for flail chest and/or complex costal fractures
Discuss thoracoscopic surgery for residual or recurrent hemothorax despite correct chest tube management
**4. Physiotherapy and mobilization**
Respiratory physiotherapy at least twice per day with incentive spirometry
Early mobilization in pain-controlled patients

9.2. Prohibited associated treatments and procedures

Sedative drugs are not allowed during NIV. However, an anxiolytic treatment may be used at a minimal effective dose to ensure patient comfort during the nocturnal periods or to treat a withdrawal syndrome.

All other aspects of patients’ clinical management remained at the attending physician’s discretion excluding the investigators.

10. CONDUCTING THE RESEARCH

10.1. THE RESEARCH SCHEDULE

- Duration of the inclusion period: **24 months**
- Participation duration of each participant: **14 days and/or end-of-hospitalization**

10.2. SUMMARY TABLE OF THE PARTICIPANT FOLLOW-UP

	Pre-inclusion	Inclusion (within 48 hours after pre-inclusion)	Every 6 hours during the first 48 hours	Daily between day 1 and day 14	End-of-hospitalization
**Information of the patient (R)**	✓				
**Inclusion and exclusion criteria (R)**	✓				
**Thoracic CT scan (C)**	✓				
**ß-HCG levels**	✓				
**Free, informed and written consent signed by the participant or legal representative and the investigator (R)**		✓			
**Randomization (R)**		✓			
**Clinical examination (C)**	✓		✓	✓	
**Arterial Gas Sampling (C and R)**	✓		✓	✓	
**Chest X rays or chest ultrasound (C)**				✓	
**Adverse events (R)**		✓	✓	✓	✓

10.3. PRE-INCLUSION VISIT

During the pre-inclusion visit, the investigator informs the participant or his/her legal representative and answers all questions regarding the purpose, nature of the constraints, foreseeable risks and the expected benefits of the research. The investigator also specifies the participant's and legal representative's rights in the context of a study and checks the eligibility criteria. A copy of the information note and the consent form is then given to the participant or his/her legal representative by the investigating doctor. After this information session, the participant or his/her legal representative has a reflection period.

The realisation of a chest CT scan is recommended as part of the standard care of blunt chest trauma patients. The realisation of blood gas analysis is also part of the standard care in hypoxemic chest trauma patients.

10.4. INCLUSION VISIT

10.4.1. COLLECTION OF CONSENT

The inclusion visit is performed by the investigator. The inclusion visit takes place within 48 hours after the pre-inclusion visit. Prior to any research-related examination, the investigator will collect the participant's or legal representative's free, informed and written consent.

This must be signed before any clinical or para-clinical examination is conducted as required by the research.

If the participant or legal representative gives his or her consent to participate, he or she and the investigator shall clearly write their names and surnames, date and sign the consent form, in two original copies. The copies of the briefing note and the consent form shall subsequently be allocated as follows:

- The investigator shall then give a copy of the briefing note and the signed consent form to the participant or legal representative.
- The investigator shall keep the other original copy (even in the event of a participant's change of address during the course of the research project) in a safe place inaccessible to third parties.

If the consent was signed by the legal representative and patient turn back in capacity to consent by himself/herself during his/her participation to the research, the investigator informs and answers all questions of

participant regarding the research. Then, participant have to confirm his/her will to continue his/her participation by signing the legal representative's consent. Otherwise participant is free to stop his/her participation.

10.4.2. Randomisation procedure

The investigator completes the randomization form on the trial e-CRF after having previously confirmed all of the eligibility criteria of the participant on the site. After validation of the content, the randomization is performed and the e-CRF gives immediately the result of the randomization (i.e. the group of randomization in particular the strategy/ procedure group allocated to the participant). The strategy randomized is setup and performed by the investigational team.

10.5. Follow-up

During the first 48 hours, will be recorded blood gas analysis (PaO_2/FiO_2 ratio, pH, $PaCO_2$), the respiratory rate and the dyspnea score measured every 6 hours.

Between Day 1 and Day 14, will be recorded daily:

- The daily duration of NIV, HFNC-O_2 or COT;
- The minimal PaO_2/FiO_2 ratio;
- The maximal respiratory rate;
- The occurrence of secondary complications (nosocomial pneumonia, secondary pleural effusion needing chest tube insertion, septic shock, cardiac arrest or severe arrhythmia, vasopressor requirement, transfusion of blood product);
- The occurrence of adverse effects of NIV or HFNC-O_2 (gastrointestinal intolerance, skin lesions, conjunctival irritation);
- The occurrence of adverse events.

10.6. End of research visit

On Day 14 or on day of hospital discharge if it occurred earlier corresponds to the term of participation to the research for patient. At this date, it will be recorded:

- The ICU and hospital length of stay;
- The status (alive or dead);
- The total duration of NIV;
- The total duration of HFNC-O_2;
- The total duration of COT;
- The need for mechanical ventilation;
- The cause of intubation;
- The total duration of mechanical ventilation;
- The occurrence of secondary complications;
- The occurrence of adverse effects of NIV or HFNC-O_2;
- The occurrence of adverse events.

10.7. Rules for stopping the research

10.7.1. End of study

It correspond to the end of the participation of the last subject (cf. Articles L.1123-11, R.1123-59 of the Code of Public Health), also called last visit of the last participant included in the study. When the study reaches its expected term, the end of the study must be reported to the ANSM within 90 days.

10.7.2. Discontinuation of research premature

This is particularly the case when the sponsor decides:

- Not to begin the research despite obtaining the permission of the ANSM and the CPP to do so;
- Not to resume the research after temporarily discontinuing it or after its suspension by the ANSM.

When the research is definitively discontinued in advance, the end of the research must be reported to the ANSM within 15 days with an explanation of the reasons for doing so.

10.7.3. Temporary discontinuation of research

The temporary interruption of clinical research (cf. Article R.1123-55 du CSP) consists of:

- Stopping the inclusion of new subjects;
- And/or discontinuation of the medical acts specified in the research protocol.

Any decision of the sponsor to this end must be informed immediately to the ANSM and the CPP. Thereafter and within a maximum of 15 days following the date of this interruption, an application for authorization to make substantial modifications to the temporary suspension must be requested from the ANSM and the opinion of the CPP must be sought.

10.8. Protocol deviations

Deviations can affect all aspects of a research protocol: inclusion, monitoring, measurement of endpoints, treatment process. All must be documented by the investigator and discussed by the Scientific Committee and Data management Centre.

Even in the event of deviation from the protocol, participants must be monitored until the date planned in the protocol.

10.8.1. Premature and definitive discontinuation of the evaluated procedure

A patient has to be considered as stopping the study when he/she no longer follows the study procedure but he/she is still followed in the study (visits, blood samplings…). Everything has to be done to collect the information of the primary criteria variable in the time lapse expected in the protocol.

Premature discontinuation of the study procedure has to be notified to the coordinating investigator. The reason for and the date of end of study procedure have to be documented.

Any patient who stops the study procedure has to be cared for as best as possible according his/her health status and medical knowledge.

10.8.2. Participants wrongly included

A participant is considered wrongly included when at least one eligibility criteria are not validated. Participants wrongly included should be discussed by the Scientific Committee.

10.8.3. Patients lost to follow up

As participants are hospitalized during all their participation, loss of follow up will not occur in this research.

10.8.4. Other deviations

The protocol deviations should be documented and justified. Violations are considered major deviations on:

- Regulatory aspects,
- The primary endpoint.

All major violations are subject to a presentation to the Scientific Committee to decide whether the patient's data can be exploited.

10.9. Constraints related to the research and possible compensation of participants

Constraints are related to the need of additional biological examinations, particularly arterial gas sampling in experimental strategy. Patients discomfort is limited by the introduction of an arterial catheter.

Patients will not receive any remuneration for their participation.

11. MANAGEMENT OF ADVERSE EVENTS AND NEW FACTS

11.1. DEFINITIONS

Adverse Event (Article R1123-46 of the Public Health Code)
Any harmful event occurring in a participant in a research study that involves human participant, whether or not the event is related to the research or product to which the research relates.

Adverse reaction (Article R1123-46 of the Public Health Code)
Any adverse event occurring in a participant in a research study that involves human participant, when this event is related to the research or product to which the research relates.

Serious adverse reaction or event (Article R1123-46 of the Public Health Code and ICH-E2B guideline)
Any adverse reaction or event that:

- ✓ results in death,
- ✓ is life-threatening,
- ✓ requires hospitalisation or prolongation of existing hospitalisation,
- ✓ results in persistent or significant incapacity/disability,
- ✓ results in a congenital anomaly or malformation,
- ✓ or any other medically important condition,

and in case of studies concerning drugs, regardless of the dose administered.

The expression “life-threatening" is reserved for an immediate life threat at the time of the adverse event.

Unexpected adverse reaction (Article R1123-46 of the Public Health Code)
Any adverse reaction of which the nature, severity, or development is inconsistent with the information on the products, procedures, and methods used in the research.

New information (Article R1123-46 of the Public Health Code)
Any new data that could lead to

- a reassessment of the benefit-risk ratio of the study of the product relating to the research,
- adjustments in the use of this product, in the conduct of the research project, or of the documents of the study,
- suspend or interrupt or modify the protocol of the research project or similar research projects.

11.2. DESCRIPTION OF EXPECTED ADVERSE EVENTS

The expected adverse events listed below are noticed as main or secondary evaluation criteria:

1. **Chest - trauma related secondary respiratory complications**

- Delayed respiratory failure requiring mechanical ventilation
- Cardiac arrest, hypotension or severe arrhythmia
- Nosocomial pneumonia, septic shock
- Secondary pleural effusion needing chest tube insertion
- Anemia and transfusion of blood product

2. **Adverse reactions of NIV or HFNC-O_2**

- Gastrointestinal intolerance (abdominal distension, nausea, vomiting, inhalation)
- Skin lesions, conjunctival irritation

Any adverse event that is not listed in the list of expected events is considered as unexpected.

11.3. Procedure to follow by the investigator in case of adverse event, new information or pregnancy

11.3.1. Reporting of adverse events (AE)

The investigator is responsible for the reporting of adverse events occurring from the date of consent is signed until the end of the patient's participation.

In this study, all the adverse events must be reported.

The investigator report adverse events in the adverse event form in the e-CRF. These adverse events are assessed at each visit during the course of the study through an interview and during the clinical examination of the patient.

11.3.2. Notification without delay of serious adverse events (SAEs) and new facts

The investigator evaluates each AE with regard to its seriousness.
The investigator must notify to the Safety and vigilance unit any serious adverse event (SAE), and any new information, **without delay** from the day he/she becomes aware of it. To report a SAE, the investigator must entirely complete and sign the SAE declaration form, before sending it via email (vigilance.essais-cliniques@chu-bordeaux.fr) or via fax (05 57 82 12 62).

If the investigator becomes aware of a **serious adverse event occurring after the end of the study** in a subject that he/she has treated and **which is possibly related to the research project,** then he/she must inform without delay the Safety and vigilance unit.

The investigator must document the event as best as he/she can and, if possible, give its medical diagnosis. As additional information becomes available, the investigator should send an updated SAE form to the Safety and vigilance unit, as soon as possible.

In addition to the SAE notification form, the investigator must send by fax or email, copies of laboratory results and examination or hospitalisation reports with information about the serious adverse event, including relevant negative results **without neglecting to anonymise these documents** and indicating the patient's number and code.

The investigator must follow each SAE until its resolution, the patient's stabilisation to a condition that he/she considers medically acceptable, or a return to the patient's previous state, even if the patient has stopped the procedure relating to the study. The investigator shall send to the Safety and vigilance unit additional information relating to the evolution of the event, if this has not been mentioned in the first report.

All serious adverse events for which the investigator or the Safety and vigilance unit considers that the event is reasonably related to the subject's involvement in the study must be suspected as being serious adverse reactions.

11.3.3. Notification of pregnancies

The occurrence of a pregnancy during or immediately following the research project shall not be considered as a SAE. However, if a woman falls pregnant within the framework of the research project, the pregnancy must be reported according to the same terms as a SAE because it shall be subject to particular follow-up until its end.

To this end, the investigator shall inform the Safety and vigilance unit using the pregnancy declaration form.

The investigator must monitor the patient until the pregnancy's end or its interruption, and inform the sponsor of either outcome. Any anomaly found in the foetus or child must be reported. Any voluntary termination of pregnancy, medical termination of pregnancy or miscarriage must be reported, and if a seriousness criteria is present it must be subject to a SAE notification. If the case involves paternal exposure, the investigator must obtain the consent of the parturient to collect information on the pregnancy.

11.3.4. Summary table of the notification circuit by event type

Event type	Notification terms	Deadline for notification to the sponsor
**Non-serious** adverse event	In the case report form	No immediate notification

Serious adverse event	Initial SAEdeclaration form (+ Follow up when necessary) + written report if necessary	**Immediate notification to** the Safety and vigilance unit
New information	Written report	**Immediate notification to** the Safety and vigilance unit
Pregnancy	Pregnancy declaration form	**As soon as pregnancy is confirmed**

Clinical Research Safety and Vigilance Unit - CHU de Bordeaux
Tel.: 05 57 82 08 34
Fax: 05 57 82 12 62
Email: vigilance.essais-cliniques@chu-bordeaux.fr

11.4. DECLARATION BY THE SPONSOR OF UNEXPECTED SERIOUS ADVERSE REACTIONS AND NEW INFORMATIONS

The Safety and vigilance unit assesses whether the serious adverse reaction is expected or unexpected based on the list of expected adverse events described in paragraph 11.2 of the protocol.

The Safety and vigilance unit will report to the competent authorities and to the ethic committee any suspected unexpected serious adverse reaction (SUSAR) occurred in France or outside the national territory within the following deadlines:

1) In the case of a SUSAR leading to death or life-threatening, without delay from the day in which the sponsor becomes aware of it,

2) In the case of other SUSARs, no later than 15 days from the day the sponsor becomes aware of them.

The Safety and vigilance unit will declare a follow-up report to French Agency of Drug Safety (Agence Nationale de Sécurité des Médicaments et des produits de santé, ANSM) for each relevant additional information concerning SUSARs:

- In the case of a SUSAR leading to death or life-threatening, the follow-up will be declared within 8 days from the declaration referred to in point 1)

- In other cases of of other SUSARs and in the event of a new fact, the relevant additional information shall be transmitted within a further period of 8 days from the deadline mentioned in point 2).

The sponsor and the investigator shall take the appropriate urgent measures. The sponsor shall inform the competent authority and the Ethical Committee of any such measures.

11.5. ANNUAL SAFETY REPORT

At the anniversary date of the first inclusion, the Safety and vigilance unit drafts a safety report that includes:

- the list of serious adverse effects potentially related to the study intervention, including both expected and unexpected serious effects, which occurred in the trial in question during the period covered by the report,
- a brief, critical analysis of the safety of study participants
- summary tables of all the serious adverse effects that occurred in the trial since the beginning of the study

The report is sent to the ANSM and the ethic committee (CPP) within 60 days of the anniversary date of the authorisation of the research project.

12. STATISTICAL ASPECTS

12.1. CALCULATION OF STUDY SIZE

The primary objective of the trial is to compare the rate of endotracheal intubation within 14 days after randomization between two NIV strategies in blunt chest trauma patients.

The documented rates of endotracheal intubation in blunt chest trauma patients with TTS score ≥ 8 and/or with indication for NIV are between 20 to 25% [5, 10-17]. A retrospective study in our institution found an endotracheal intubation rate of 12% using the strategy early NIV + HFNC-O2 [31].

Our hypothesis is that the strategy associating early NIV + HFNC-O2 may reduce from 25% to 12% the intubation rate in blunt chest trauma patients. With Chi-Square test, 139 patients per group are required to test this hypothesis, with a two-sided type I error rate of 5% and power of 80%.

12.2. STATISTICAL METHODS EMPLOYED

12.2.1. ANALYSIS STRATEGY

The study data will be analysed by the biostatistician of the Methodology and Data Management Centre (USMR, Bordeaux University Hospital).

An intention-to-treat analysis will be performed as the principal analysis, i.e. all randomised patients will be included in the analysis in the group in which they first were randomised and all their data will be used, regardless of any changes of procedure over the study duration. The missing data for the primary criterion will be replaced by a failure of the procedure (“missing = failure”). To check the robustness of the results of the ITT analysis, sensitivity analysis will be performed using first the maximum bias strategy and secondly available data. Then, an “under treatment analysis” of the available data will be performed, i.e. all randomised patients will be included in the analysis in the group of the procedure they have really exposed. A sub-group analysis will also be conducted.

Descriptive analysis will always be presented overall and by treatment group. Comparative analysis between procedure groups will be systematically performed before and after adjustment on baseline prognostic factors whose distribution could be, despite the randomization, unbalanced between procedure groups. These adjustments will potentially require using the appropriate models, whose relevance will be discussed according to the distribution and to the type of variables.

All comparisons will be performed with a type I error of 5%.

12.2.2. DESCRIPTIVE STATISTICAL METHODS

Qualitative variables will be described by frequencies and proportions. Quantitative variables will be described with frequencies, mean, standard deviation, median, range and interquartile range. We will attempt as much as possible to associate a graphic representation of the analyses.

12.2.3. CALCULATION RULES IN THE STATISTICAL ANALYSES

- Duration between two dates in months $= \dfrac{(\text{Date } 2 - \text{Date } 1 + 1)}{30.4375}$
- Duration between two dates in years will be calculated as number of years between the two dates. “YRDIF” function of SAS will be used with “Actual” option (this option deals with leap years).
- Duration between two dates in days will be calculated as the number of days between the two dates. “DATDIF” function of SAS will be used.
- Difference between two measurements will be calculated according to the following formula:
  - Delta = (measurement at time 2 – measurement at time 1)
- Age at the inclusion will be calculated as the number of entire years between inclusion date and birth date. « YRDIF » function of SAS will be used with “Actual” option (this option deals with leap years).

12.2.4. Software used

Statistical analyses will be performed with the SAS® software (version 9.4).

12.3. Analysis plan

12.3.1. Description of the inclusions and follow up

The number of patients included, the inclusions curve (progression of the number of patients included between the first and the last inclusion), the number of visits actually performed will be described for each group.

12.3.2. Patients included in the analysis

Only the patients presenting at least one of the following conditions can be excluded from the analysis:

- patients wrongly included for unsigned consent;
- patients wrongly included for major non-respected eligibility criteria;
- patients who withdrew their informed consent.

The Scientific Committee will make this decision of exclusion after documentation of observations by the Methodology and Data Management Centre, blinded to the procedure group and to the patient's evolution after inclusion.

Except for these exclusions, the patients who die, are lost to follow-up or leave the study, will all be included in the analysis.

12.3.3. Baseline characteristics

The participants will be described at baseline and during the follow-up according to the following variables:

- Demographics
- Medical history
- Clinical data
- Treatments
- Adverse events
- Deviations
- Deaths

12.3.4. Primary endpoint

The comparison between groups focuses on the proportion of patients who required endotracheal intubation within 14 days after randomization.

These 2 proportions will be compared with a Chi² test or with Fisher's exact test, according to the size of the expected values under the hypothesis of independence.

Logistic regression model will be used to adjust on stratification factor and other major confounding factors. Assumption of the models (log-linearity of the associations) will be systematically checked.

12.3.5. Secondary endpoint(s)

The comparison between groups included in the subgroup analysis (concerning only the patients having PaO2/FiO2 < 200 mmHg at inclusion) will be compared with a Chi² test or with Fisher's exact test, according to the size of the expected values under the hypothesis of independence.

Logistic regression model will be used to adjust on stratification factor and other major confounding factors. Assumption of the models (log-linearity of the associations) will be systematically checked.

Qualitative variables will be compared using the Chi-square test, the corrected Chi-square test, or the exact Fisher test according to the expected values under the hypothesis of independence. To adjust on stratification factor and other major confounding factors, a logistic or multinomial regression model will be used. The conditions of validity of these models (log-linearity of the association) will be systematically checked.

Quantitative variables will be compared using the Student t-test if the conditions of validity are respected (normal distribution, homogeneous variances). If the variances are different between both groups, the Student t-test for unequal variances will be used. In the event of non-normal distribution, the Mann-Whitney-Wilcoxon test will be used. To adjust on stratification factor and other major confounding factors, a linear regression model will be used. The conditions of validity of this model (normal distribution, homogeneous variances, linearity of the associations) will be systematically checked. Transformation of the criteria could be considered.

The longitudinal analyses of the quantitative variables will use mixed regression models if assumptions are fulfilled. The “longitudinal analyses” of the qualitative variable will use a Chi² test for trend.

A detailed analysis plan will be defined before data being locked and will have to be validated by the scientific committee of the study.

13. MONITORING OF THE RESEARCH

13.1. Scientific Committee

13.1.1. Composition

The committee is composed of the following individuals: Pr Matthieu BIAIS (President), Dr Cédric CARRIE, Dr Antoine BENARD (methodologist), Olivier BRANCHARD, Roxane COUERON (statistician), Dr Caroline ROUSSILLON ‘Clinical research safety and vigilance unit) and a representative of the sponsor.

13.1.2. Frequency of meetings

The Scientific Committee meet as necessary and at least once a year until the end of the study.

13.1.3. Role

- To take any decision motivated by the coordinating investigator, regarding the respect of the protocol and the appropriate course of the study.
- To verify the respect of ethical requirements.
- To collect information about the course of the study, potential difficulties and preliminary results asking the coordinating investigator and the Methodology and Data management Centre.
- To decide on any modification on the protocol version:
    - To facilitate recruitment of patients,
    - The amendments on the initial version of the protocol before the examination of ethics committee and the authorization of the French Health Products Safety Agency,
    - Opening or closing of investigational sites,
    - To ensure the highest safety level possible for the participants,
    - To discuss the results and the publication strategy.
- To propose to prolong or interrupt the course of the research in the event of low level of recruitment, too many patients lost to follow up, too many major protocol deviations or for legal reasons. It sets out the requirements for the long-term follow-up of the patients included in the study.
- To analyse and validate any proposition of new unscheduled biologic projects on the material of the research. The scientific committee has to define the requirements for information of the patients and the rules of data availability.
- At the end of the meeting, the president of the scientific committee has to inform the sponsor about the decisions. Decisions about major amendments or financial issues have to be approved by the sponsor.

13.2. Independent data safety monitoring committee

This research doesn’t aim to evaluate devices (NIV or HFNC-O2) which are used routinely in intensive care settings. Consequently, Independent Monitoring Committee is not necessary in this study.

13.3. Adjudication Committee

13.3.1. Composition

The committee is composed of three ICU physicians independent from the investigation centres, designated before the start of the study.

13.3.2. Frequency of meetings

Every 20 monitored cases of endotracheal intubation within 14 days after randomization.

13.3.3. Role

The independent adjudication committee will have to validate all endotracheal intubation, based on clinical, biological, and imaging data. In particular, they will have to verify the consistency of indications, based on criteria presented in chapter 4.1. This validation will be made blinded from the randomisation group. The consistency of indications will be stated at the majority of the three clinicians.

14. RIGHTS OF ACCESS TO DATA AND SOURCE DOCUMENTS

14.1. ACCESS TO DATA

Agreeing to participate in the protocol implies that the investigators will make the documents and personal data that are strictly necessary for the monitoring, quality control and auditing of the research, available in accordance with the laws and regulations in force.

14.2. SOURCE DATA

All information contained in original documents, or in authenticated copies of these documents, relating to clinical examinations, observations or other activities conducted as part of a research study and necessary for the reconstitution and evaluation of the research. The documents in which the source data are saved are called the source documents.

14.3. DATA CONFIDENTIALITY

In accordance with the legislative provisions in force, persons having direct access to source data will take all the necessary precautions to ensure the confidentiality of information relating to investigational medicinal products, research, participants, especially as regards their identity and the results obtained. These people, like the investigators, are subject to professional secrecy.

During or at the end of the research, the data collected on the participants and sent to the sponsor by the investigators (or any other specialised contributor) will made anonymous. The data must never explicitly mention the names of the persons concerned or their addresses.

Each patient will be assigned a confidential identification code, consisting of the number of the investigation centre (2 digits) and a patient number (4 digits) and a code letter (4 letters). The code letters may be constructed from the initials of the name and surname of the patient.

The sponsor will ensure that each participant has given his/her written agreement for access to the individual data concerning them and strictly necessary for the quality control of the research.

15. QUALITY CONTROL AND QUALITY ASSURANCE

15.1. GUIDELINES FOR COLLECTING DATA

All the information required by the protocol must be recorded in case report forms and an explanation must be provided for any missing data. The data must be collected as and when they are obtained, and transcribed in these notebooks in a clear and legible way.

Data are collected on electronic case report form (eCRF).

15.2. QUALITY CONTROL

A clinical researcher appointed by the sponsor will regularly visit each centre investigator, during the implementation of the research, one or more times during research according to the frequency of the inclusions and at the end of the research. During these visits and in accordance with the risk-based monitoring plan (participant, logistics, impact, resources), the following elements will be reviewed:

- Informed consent;
- Compliance with the research protocol and the procedures defined therein;
- Quality of the data collected in the case report form: accuracy, missing data, consistency of the data with the source documents (medical records, appointment books, originals of laboratory results, etc.);
- Management of potential products.

All visits will be the subject of a monitoring report by written report.

15.3. DATA MANAGEMENT

15.3.1. SOFTWARE USED

The software used for data management is REDCap. It is coupled with a dedicated MySQL database.

15.3.2. DATA HOSTING

The Information Systems Department (DSI) of the CHU of Bordeaux provides hosting and maintenance of the database in accordance with its procedures. The servers (Internet and database) are housed in the premises of the DSI (Talence, Gironde).

15.3.3. DATA SECURITY

A backup server for the server of the CHU of Bordeaux is also hosted in Roubaix (59) and a second is in a remote site located in Gravelines (59).

The management of the access's rights to the software is under the responsibility of the REDCap administrator of the CHU of Bordeaux .

15.3.4. CRF BUILDING

The eCRF input masks are based on a WORD version of the CRF, which is written by the coordinating CRA. The final validation of the document is done by the data manager. A replay circuit defined in the standard booklet validates the CRF. Once the CRF is validated, the data manager creates the input masks.

15.3.5. DATA ENTRY

Data entry into the eCRF is the under the responsibility of the investigator's center. Anyone other than the investigator conducting the seizure in the eCRF must be previously trained and delegated by the investigator to do so. The eCRF is developed via the REDCap software by the data manager of the study.

15.3.6. ACCESS RIGHTS

Only database managers, DMC project team and auditors have access's rights to the database.

15.3.7. ROLES

The Data Manager is responsible for the queries. The Data manager has to execute batches to update the querie's list. The investigator's team is responsible for data updates. No member of the DMC project team shall replace the investigator's team in data corrections. The CRA has to code adverse events and treatments under investigator's responsibility. The Data manager is responsible for managing querie batches. Querie batches should be run regularly by the data manager.

15.3.8. DATA CODING

The prescribed treatments and clinical events are coded in the eCRF in order to carry out data testing and data analysis.

The following dictionaries are used for the coding of medical terms:

- MedDRA (current version) FR/US
- ATC version

15.3.9. DATA TESTING

Data controls are made to validate data consistency and the presence of mandatory data. Data controls'slist is defined jointly by the coordinator investigator with the DMC, and is reported in the study's DataValidation Plan.

Queries are created and managed by the data manager. They are sent to the investigator's team via REDCap. The investigator's team do the data correction required to solve the queries.

15.3.10. AE/SAE RECONCILATION

If necessary, the crossing of the bases is carried out by the Security and Vigilance Unit of the CHU of Bordeaux according to the procedure in force. The reconciliations are carried out regularly according to a rhythm defined by the Independent Monitoring Committee. The Data Manager is responsible for forwarding the adverse event's table to the USV. The data are exported as an Excel file which is submitted on the CIRRUS secure exchange platform.

15.3.11. Database freezing

The database shall be frozen for the final analysis. The database freezing process is carried out in accordance with the DMC SOP in place.

15.3.12. Data transfer

If necessary, data transfer can be considered. Data transfers (sending, receiving) are carried out in accordance with the procedure in force at the CMG and according to the desired formats according to existing constraints. For security reasons, the data files are anonymized and transferred via the secure platform CIRRUS.

15.3.13. Archiving the database

Study's sponsor is responsible of the database archiving. Study's data are stored on the DSI server and a physical copy is kept by the sponsor.

15.3.14. Quality management

The activities of the CMG project team are integrated in the Quality Management System. Non-compliance management is ensured in accordance with the CMG SOP in place.

15.4. Audit and inspection

Individuals appointed by the sponsor and independent of those conducting the study may carry out audits at any moment. The audit is designed to check the safety of the participants, the respect of their rights, the compliance with applicable regulations and data reliability.

An inspection may also be undertaken by a competent authority (ANSM in France or EMA within the framework of a European study, for example).

The audit, as well as the inspection, may be applied to any stage of the research, from protocol development to the publication of results and the classification of data used or produced as part of the research project.

The investigators shall comply with the sponsor's requirements regarding the audit and the competent authority's inspection of the research.

16. ETHICAL AND REGULATORY CONSIDERATIONS

The sponsor and the investigator(s) undertake to ensure that this research is carried out in accordance with law no. 2012-300 of 5 March 2012 on research involving the human person, as well as in agreement with Good Clinical Practices (ICH version 4 of 9 November 2016 and the decision of 24 November 2006) and the Declaration of Helsinki (which can be found in full athttp://www.wma.net).

The research is conducted in accordance with this protocol. Except in emergency situations that require the implementation of specific therapeutic acts, the investigator(s) undertake(s) to respect the protocol in all points especially with regard to the collection of consent and notification and follow-up of serious adverse events.

This research received a favourable opinion from the Committee for the Protection of Persons (CPP) and authorisation from the ANSM.

The CHU de Bordeaux, sponsor of this research, has taken out a civil liability insurance contract with HDI GLOBAL SE in accordance with the provisions of the Public Health Code.

The data recorded during this research are the subject of a computerised processing at USMR - CHU of Bordeaux in accordance with the law on data processing, files and liberties (no. 78-17 of 6th January 1978 as amended by Law 2018-493 of 20th June 2018) and GDPR (Regulation EU 2016/679).

This research falls within the framework of the "Reference Methodology" (MR-001) in application of the provisions of Article 54 paragraph 5 of the amended law of 6 January 1978 relating to information, files and liberties.. The USMR - CHU of Bordeaux has signed a compliance commitment to this "Reference Methodology".

This research has been registered on the site http://clinicaltrials.gov/

Changes to the protocol

Any substantial change, i.e. any change that is likely to have a significant impact on the protection of persons, on the conditions of validity and on the results of the research, on the quality and safety of the products tested, on the interpretation of scientific documents that support the conduct of the research or the way in which the research is conducted, is subject to a written amendment submitted to the sponsor. The latter must obtain, prior to its implementation, a favourable opinion from the CPP, and, where applicable, authorisation from the French National Agency for Medicines and Health Products Safety (ANSM).

Non-substantial changes, i.e, those that do not have a significant impact on any aspect of the research, are communicated to the CPP for information.

All changes are validated by the sponsor, and by all research stakeholders involved in the change, before submission to the CPP, and, where applicable, to the ANSM. This validation may require the meeting of all committees formed for the research. .

All changes to the protocol must be made known to investigators, who are participating in the research. The investigators undertake to respect the content.

Any modification that modifies participant care or the benefits, risks and constraints of the research is the subject of a new information note and a new consent form whose collection follows the same procedure above.

17. PRESERVATION OF RESEARCH DOCUMENTS AND DATA

The following documents related to this research are archived by the investigator in accordance with Good Clinical Practices

- for a period of **15 years following the end of the research** :
  - The protocol and any modifications to the protocol
  - Case report forms (copies)
  - Source records of participants who have signed a consent form
  - All other documents and correspondence related to research
- for a period of **30 years following the end of the research**:
  - The original copy of the informed consent forms signed by the participants

All of these documents are the responsibility of the investigator during the regulatory archiving period. No displacement or destruction can be made without the agreement of the sponsor. At the end of the regulatory archiving period, the sponsor will be consulted for destruction. All data, documents and reports may be subject to audit or inspection.

18. FINAL REPORT

Within one year after the end of the research or its interruption, a final report will be prepared and signed by the sponsor and the investigator.

This report will be kept at the disposal of the competent authority.

The sponsor will transmit to the CPP and, where applicable, to the ANSM, the results of the research in the form of a summary of the final report within one year after the end of the research.

19. RULES FOR PUBLICATION

19.1. Scientific communications

The data analysis provided by the investigating centres is carried out by USMR - CHU of Bordeaux. This analysis gives rise to a written report which is submitted to the sponsor, who will forward it to the Committee for the Protection of Persons and to the competent authority.

Any written or oral communication of the results of the research must have the prior consent of the coordinating investigator and, where appropriate, of all committees that were established for the research.

The coordinating investigator undertakes to make available to the public both negative and inconclusive and positive research results.

The publication of the main results must mention the name of the sponsor, all the persons who helped in the inclusion or follow-up of participants in the research, methodologists, biostatisticians and data managers who participated in the research, health vigilance personnel who participated in the safety analysis of the participants and members of the committee(s) constituted for the research. International rules of writing and publication will be taken into account (The Uniform Requirements for Manuscriptsof the ICMJE, April 2010).

19.2. Communication of results to participants

In accordance with law no. 2002-303 of 4 March 2002, the participants are informed, at their request, of the overall results of the research.

19.3. Transfer of data

Data management is provided by USMR - CHU of Bordeaux. The conditions for the transfer of all or part of the research database are decided by the research sponsor are the subject of a written contract.

20. BIBLIOGRAPHIC REFERENCES

1. Michelet P, Boussen S. Case scenario - thoracic trauma. Ann Fr Anesth Reanim. 2013;32(7-8):504-9.
2. Unsworth A, Curtis K, Asha S. Treatments for blunt chest trauma and their impact on patient outcomes and health service delivery. Scand J Trauma Resusc Emerg Med. 2015; 23(1):17.
3. Blecher GE, Mitra B, Cameron PA, Fitzgerald M. Failed Emergency Department disposition to the ward of patients with thoracic injury. Injury 2008;39:586– 91.
4. Mommsen P, Zeckey C, Andruszkow H, Weidemann J, Frömke C, Puljic P, van Griensven M, Frink M, Krettek C, Hildebrand F. Comparison of different thoracic trauma scoring systems in regards to prediction of post-traumatic complications and outcome in blunt chest trauma. J Surg Res. 2012;176(1):239-47
5. Daurat A, Millet I, Roustan J-P, Maury C, Taourel P, Jaber S, et al. Thoracic Trauma Severity score on admission allows to determine the risk of delayed ARDS in trauma patients with pulmonary contusion. Injury. 2016;47(1):147-53
6. Antonelli M, Moro ML, Capelli O, De Blasi RA, D'Errico RR, Conti G, Bufi M, Gasparetto A. Risk factors for early onset pneumonia in trauma patients. Chest. 1994;105(1):224-8.
7. Beitler JR, Malhotra A, Thompson BT. Ventilator-induced Lung Injury. Clin Chest Med. 2016;37(4):633-646.
8. Dres M, Goligher EC, Heunks LMA, Brochard LJ. Critical illness-associated diaphragm weakness. Intensive Care Med. 2017. doi: 10.1007/s00134-017-4928-4
9. Alisha C, Gajanan G, Jyothi H. Risk Factors Affecting the Prognosis in Patients with Pulmonary Contusion Following Chest Trauma. J Clin Diagn Res. 2015;9(8):17-9.
10. Chiumello D, Coppola S, Froio S, Gregoretti C, Consonni D. Noninvasive ventilation in chest trauma: systematic review and meta-analysis. Intensive Care Med. 2013; 39(7):1171-80.
11. Ferrer M, Esquinas A, Leon M, Gonzalez G, Alarcon A, Torres A. Noninvasive ventilation in severe hypoxemic respiratory failure: a randomized clinical trial. Am J Respir Crit Care Med. 2003; 168(12):1438-44.
12. Hernandez G, Fernandez R, Lopez-Reina P, Cuena R, Pedrosa A, Ortiz R, et al. Non-invasive Ventilation Reduces Intubation in Chest Trauma-Related Hypoxemia: A Randomized Clinical Trial. Chest 2010; 137:74–80.
13. Bolliger CT, Van Eeden SF. Treatment of multiple rib fractures. Randomized controlled trial comparing ventilatory with nonventilatory management. Chest 1990; 97:943–8.
14. Gunduz M, Unlugenc H, Ozalevli M, Inanoglu K, Akman H. A comparative study of continuous positive airway pressure (CPAP) and intermittent positive pressure ventilation (IPPV) in patients with flail chest. Emerg Med J 2005; 22:325–9.
15. Roberts S, Skinner D, Biccard B, Rodseth RN. The role of non-invasive ventilation in blunt chest trauma: systematic review and meta-analysis. Eur J Trauma Emerg Surg. 2014; 40(5):553-9.
16. Duggal A, Perez P, Golan E, Tremblay L, Sinuff T. The safety and efficacy of noninvasive ventilation in patients with blunt chest trauma: a systematic review. Crit Care 2013; 17:R142.
17. Beltrame F, Lucangelo U, Gregori D, Gregoretti C. Noninvasive positive pressure ventilation in trauma patients with acute respiratory failure. Monaldi Arch Chest Dis. 1999;54(2):109-14.
18. Bouzat P, Raux M, David JS, Tazarourte K, Galinski M, Desmettre T, Garrigue D, Ducros L, Michelet P; Expert's group, Freysz M, Savary D, Rayeh-Pelardy F, Laplace C, Duponq R, Monnin Bares V, D'Journo XB, Boddaert G, Boutonnet M, Pierre S, Léone M, Honnart D, Biais M, Vardon F. Chest trauma: First 48hours management. Anaesth Crit Care Pain Med. 2017;36(2):135-145.
19. Rochwerg B, Brochard L, Elliott MW, Hess D, Hill NS, Nava S, Navalesi P Members Of The Steering Committee, Antonelli M, Brozek J, Conti G, Ferrer M, Guntupalli K, Jaber S, Keenan S, Mancebo J, Mehta S, Raoof S Members Of The Task Force. Official ERS/ATS clinical practice guidelines: noninvasive ventilation for acute respiratory failure. Eur Respir J. 2017; 50(2).
20. Mauri T, Turrini C, Eronia N, Grasselli G, Volta CA, Bellani G, Pesenti A. Physiologic Effects of High-Flow Nasal Cannula in Acute Hypoxemic Respiratory Failure. Am J Respir Crit Care Med. 2017; 195(9):1207-1215.
21. Vargas F, Saint-Leger M, Boyer A, Bui NH, Hilbert G. Physiologic Effects of High-Flow Nasal Cannula Oxygen in Critical Care Subjects. Respir Care. 2015; 60(10):1369-76.
22. Rittayamai N, Tscheikuna J, Praphruetkit N, Kijpinyochai S.Use of High-Flow Nasal Cannula for Acute Dyspnea and Hypoxemia in the Emergency Department. Respir Care. 2015;60(10):1377-82.
23. Stéphan F, Barrucand B, Petit P, Rézaiguia-Delclaux S, Médard A, Delannoy B, Cosserant B, Flicoteaux G, Imbert A, Pilorge C, Bérard L; BiPOP Study Group. High-Flow Nasal Oxygen vs Noninvasive Positive Airway Pressure in Hypoxemic Patients after Cardiothoracic Surgery: A Randomized Clinical Trial. JAMA. 2015; 313(23):2331-9.
24. Frat JP, Thille AW, Mercat A, Girault C, Ragot S, Perbet S, Prat G, Boulain T, Morawiec E, Cottereau A, Devaquet J, Nseir S, Razazi K, Mira JP, Argaud L, Chakarian JC, Ricard JD, Wittebole X, Chevalier S, Herbland A, Fartoukh

M, Constantin JM, Tonnelier JM, Pierrot M, Mathonnet A, Béduneau G, Delétage-Métreau C, Richard JC, Brochard L, Robert R; FLORALI Study Group; REVA Network. High-flow oxygen through nasal cannula in acute hypoxemic respiratory failure. N Engl J Med. 2015; 372(23):2185-96
25. Mosier JM, Sakles JC, Whitmore SP, Hypes CD, Hallett DK, Hawbaker KE, Snyder LS, Bloom JW. Failed noninvasive positive-pressure ventilation is associated with an increased risk of intubation-related complications. Ann Intensive Care. 2015; 5:4.
26. Carrillo A, Gonzalez-Diaz G, Ferrer M, Martinez-Quintana ME, Lopez-Martinez A, Llamas N, Alcazar M, Torres A. Non-invasive ventilation in community-acquired pneumonia and severe acute respiratory failure. Intensive Care Med. 2012 Mar;38(3):458-66. doi: 10.1007/s00134-012-2475-6. Epub 2012 Feb 9.
27. Esteban A, Frutos-Vivar F, Ferguson ND, Arabi Y, Apezteguía C, González M, Epstein SK, Hill NS, Nava S, Soares MA, D'Empaire G, Alía I, Anzueto A. Noninvasive positive-pressure ventilation for respiratory failure after extubation. N Engl J Med. 2004 Jun 10;350(24):2452-60.
28. Antonelli M, Conti G, Moro ML, Esquinas A, Gonzalez-Diaz G, Confalonieri M, et al. Predictors of failure of noninvasive positive pressure ventilation in patients with acute hypoxemic respiratory failure: a multi-center study. Intensive Care Med 2001; 27:1718–28
29. Gay PC. Complications of noninvasive ventilation in acute care. Respir Care. 2009;54(2):246-58
30. Hill NS. Complications of noninvasive ventilation. Respir Care 2000; 45 (5) : 480-1
31. Carrie C, Stecken L, Cayrol E, Cottenceau V, Petit L, Revel P et al. Bundle of care for blunt chest trauma patients improves analgesia but increases rates of intensive care unit admission: A retrospective case-control study. Anaesth Crit Care Pain Med. 2018;37(3):211-215.
32. Michelet P, Couret D, Brégeon F, Perrin G, D'Journo X-B, Pequignot V, et al. Early onset pneumonia in severe chest trauma: a risk factor analysis. J Trauma. févr 2010;68(2):395‑400
33. Leone M, Bouadma L, Bouhemad B, Brissaud B, Dauger S, Gibot S, Hraiech S, Jung B, Kipnis E, Launey Y, Luyt C.E, Margetis D, Michel F, Mokart D, Montravers P, Monsel A, Nseir S, Pugin J, Roquilly A, Velly L, Zahar J.R, Bruyère R, Chanques G. Recommandations formalisées d'experts. Pneumonies associées aux soins de réanimation. 2017
34. Kalil AC, Metersky ML, Klompas M, Muscedere J, Sweeney DA, Palmer LB, Napolitano LM, O'Grady NP, Bartlett JG, Carratalà J, El Solh AA, Ewig S, Fey PD, File TM Jr, Restrepo MI, Roberts JA, Waterer GW, Cruse P, Knight SL, Brozek JL. Management of Adults With Hospital-acquired and Ventilator-associated Pneumonia: 2016 Clinical Practice Guidelines by the Infectious Diseases Society of America and the American Thoracic Society. Clin Infect Dis. 2016; 63(5):e61-e111.
